# The Racial and Social Incidence of Cancer of the Uterus

**DOI:** 10.1038/bjc.1948.26

**Published:** 1948-09

**Authors:** E. L. Kennaway


					
BRITISH JOURNAL OF CANCER

VOL. II        SEPTEMBER, 1948          NO. 3

THE RACIAL AND SOCIAL INCIDENCE OF CANCER

OF THE UTERUS.
E. L. KENNAWAY.

From the Pathological Department, St. Bartholomew's Hospital,

London, E.C. 1.

Received for publication, July 5, 1948.

RACIAL INCIDENCE.

A. Upon Jewish Women:

London.
Munich.
Vienna.

Budapest.

Amsterdam.
Rotterdam.
Sweden.
Paris.

Palestine.

New York.

Mayo Clinic.
Chicago.

New York, Chicago and Philadelphia.
Summary.

B. Upon Hindu, Moslem, Parsee and Indian

Christian Women.

c. Upon Chinese Women.

TENTS.

SOCIAL INCIDENCE.

Munich-Bavaria.

England and Wales.
U.S.A., France.

FACTORS WHICH MAY AFFECT THE INCIDENCE

OF CANCER OF THE UTERUS.

1. Ritual Observances and Ablutions asso-

ciated with Menstruation and Child-birth.

(a) Jewish Ritual.
(b) Moslelm Law.

(c) Parsee Ritual.
(d) Hindu Ritual.
2. Economic Conditions.
3. Child-bearing.

4. Early Marriage.

5. Genetic Factors.
6. Circumcision.
7. Douching.

CANCER OF THE CORPUS UTERI.
DISCt SSION.
SUMMARY.

THE following paper serves to show the very inadequate state of our know-
ledge in a field where abundant clinical material is available for investigation.
An attempt has been made to collect and summarize the very scattered literature
and to save others the task of examining the actual papers. Some of the authors
misrepresent their own data, misquote those of others, are defective in arithmetic
and make the task of anyone who wishes to examine their conclusions difficult.
Some authors, when giving data for the incidence of cancer other than uterine,
do not say whether the figures refer to one or both sexes; and in statistics based
on admissions to a hospital they do not say whether this is a women's hospital
or a general hospital, or where the hospital is situated, data which are not always
easy to obtain in another country. One hopes that this presentation of the
literature may lead to further investigation of questions, such as the social
incidence of cancer of the cervix in this country, upon which more information
should be available.

13

E. L. KENNAWAY

RACIAL INCIDENCE.

(A) Upon Jewish Women.

The earliest statements on this subject are not based on statistical material.
Braithwaite (1901) says,"    . . . cancer of the uterus was seldom or never
met with amongst the numerous Jewesses attending the gynaecological out-
patient department of the Leeds General Infirmary (only one case in 10 years).
The experience of the London Hospital where there is a special Hebrew depart-
ment, is the same (only one case in five years against 178 amongst Gentile
women)."
London.

The number of Jews in London is unknown and no official British statistics
give data for Jews separately. Sorsby (1931) utilized the records of Jewish
burial societies.  He says,   . . . the Jewish community possesses a practi-
cally complete register of its deaths. This comes about through the extreme
rarity of a Jew, whatever his mode of life, being buried in a non-Jewish cemetery."
He based his study of cancer in Jews upon 20,422 entries during 1910-1925 in
the books of the Burial Society of the United Synagogues; the reasons for this
restriction of the field are given in full in his book. All records of deaths from
cancer were checked by comparison with the corresponding death certificates at
the General Register Office. In Table I a comparison is made with data for the
whole of England and Wales.

TABLE I.-Deaths from     Cancer of Uterus.   (Sorsby.)

Total deaths of  Deaths from cancer

1911-1920.            women from cancer.    of uterus.   B per cent of A.

A.                B.

England and Wales        .     .    224,503      .     40,175     .     17 9
Jews of London      .    .     .        550      .         58     .     10 5
M3unich.

Theilhaber (1910) gives the data shown in Table II on the authority of H.
Kirschner, and states that cancer of the uterus makes up 25 to 35 per cent of
cancers* in the general population of women, in contrast to the 7 per cent shown
by these figures for Jewesses. The age distribution of these 7 cases was as
follows: 30-40, 1; 40-50, 2; 50-60, 2; 60-70, 2.

The seven cases of uterine cancer in Jewesses which appear in Table II appear
to be identical with those in Table III.

* This is a very high percentage, for which Theilhaber shows no detailed evidence. The Registrar-
General's Statistical Review (1935-1939) gives the following figures for England and Wales for the
five years 1935-1939:

Deaths of women from all forms of malignant disease  .  .  .  .  .  . 175,366
Deaths from malignant disease of the uterus  .  .  .  . 22,303 or 12.7 per cent.
The corresponding figure given by Fischberg (Table XI) for a largely non-Jewish population,
apparently of women onlly, is 16 per cent. See also the data from Amsterdam (Table VII; 14 per
cent), and from the Mayo Clinic (Table XVII; 16-5 per cent). However, Theilhaber and Greischer
(Table III) give a figure (28.7 per cent) from Munich in the later years (1907-09) of the period dealt
with by Theilhaber which confirms his statement.

178

INCIDENCE OF CANCER OF UTERUS

TABLE II.-Deaths of Jews from Cancer, Munich, 1876-1908. H. Kirschner,

published by F. Theilhaber, 1910.)

Males.    Females.
Deaths of Jews from cancer  .     .    .    87    .    98
Cancer of uterus  .    .    .     .    .          .     7
Cancer of stomach and intestines  .    .    -     .    41

Theilhaber and Greischer (1910) record the number of deaths from cancer in
Munich (1) in the whole population of persons aged 25 and over in 3 years,
1907-1909 (A); (2) in the Jewish population in 19 years, 1880-1908 (B).

Figures from A are given under "Christians" in their Table XIII, though
one is not told whether the "whole population" of A includes Jews; but the
number of Jewish deaths in 3 years might not introduce any serious error. In
the same table, figures from the three years (1907-1909) of A are placed under the
heading "1880-1908" with those of B. In spite of these careless errors, the
data seem to be of some value and are summarized in Table III, which shows that
cancer of the uterus formed a much higher percentage (28.7) of all cancers in
the general population than among Jews (6.8), while cancer of the breast showed
a difference in the opposite sense but of smaller amount (9.0 and 16.7).

TABLE III.-Cancer of the Uterus and Mamma. Munich (Theilhaber and Greischer,

1910).

Christians (1907-1909).

A~~~~~~~~~~~~~~~~~~~~~

Total cancers

Total oancers         Uterus.                  Mamma.

in women.              Per cent of total.  -      Per cent of total

1326    .    381     .    28'7    .    120     .    9'0

Jews (1880-1908).

Total cancers          Uterus.                  Mamma.

in women.               Per cent of total.        Per cent of total.

102    .     7     .      6-8    .     17     .    16'7

Population.      Jews.           Total.

1880     .    4,144     .    230,023
1905    .     10,056    .    538,983

The data (Table IV) from his own patients given by A. Theilhaber of Munich
(1909) show that the ratio of cases of myoma to those of cancer of the cervix
was, in the whole series of 5848 patients, 228 : 133 or 1I7: 1, while among
Jewesses the ratio was 43: 1. These ratios do not, of course, exclude a high
incidence of myoma upon Jewesses.

Vienna.

Peller (1931) examined the official statistics (no exact reference is given) of
Vienna, where the Jewish population numbered 200,000, and gives data upon
which Table V is based. He states that similar differences are shown in each

179

E. L. KENNAWAY

ti?

CO -

o0

o~
aq

XUMoUIl notdO

si11noqe     x 11

*oldma 'SWIsiaooMitr IIu
? u~ou)un uoldodnoav  O

?s,   SImoqWI i9naliV r - o

? ssosnoq~"I  I 0~

9OldAml 'sjxouo.p Ipnud   4

i?p~   ' sepJ   to)[.to,~t r

I co
uvqajua 's01doisoaonuu  ut

Ou 'SUJll~OWo aooI 1

*        0

?s.xa.~Sl 'slauo'l!zxl tI

I,ouaemuV.IpUOlWA" . ORO a  10

0

0

C4-4~~~~~~~~~~~~C-

,,uosqno!azssaommo~[  ?,, ~

CP    0
0

o~~~

1   .  .

o~~~~

ccaua9^l   ?>X    ,u

*

180

0

.4

?

-4
*0)

c)

0)
o

~0

I.

H:

E--

cq',~
O o
CO ~O
_~I _4

.. co
0e.,O

010

01
0 ~O
*1-1

10

*4-

0I

o
0

o_
*g

*sa;aA

csli

INCIDENCE OF CANCER OF UTERUS

one of the four years in question. Cancers of the uterus made up three-quarters
of the cancers of the female genital tract in non-Jewish women, but less than
one-half of these tumours in Jews, while cancers of the ovary provided one-half
of the Jewish genital cancers and only 18 per cent in other women. Jewesses
provide 7.63 of all genital cases, 18.4 per cent of cancers of the ovary and only
4*95 per cent of cancers of the uterus.

Ovarian tumours tend to occur at an earlier age than does uterine cancer,
but Peller says that there was no difference in age distribution between the two
groups which would introduce this factor. He states that the menarche occurs
much earlier in Jewish women than in "the whole female population," but
gives no numerical data.

TABLE V.-Deaths from Cancer of the Female Genital Tract.  Vienna* 1926-1929.

(Peller, 1931.)

Vagina.            Uterus.            Ovary.

Deaths.  Per cent  Deths    Per cent  Deaths. Per cent  Total.

of total.  eas.    of total.         of total.

Jewish   .     3  .   2    .    76  .   48    .   79   .   50   .    158
Non-Jewish.   106   .   6    .  1459  .   76    .  350   .   18   .  1915

* Davidsohn (1939) states that these data are from the Hadassah Hospital, Jerusalem, but Peller's
table is headed quite clearly "In den Jahren 1926-1929 starben in Wien an Krebs."

Budapest.

Theilhaber (1910) gives figures, summarized in Table VI, and says that they
are taken from the "Statistische Jahrbiucher der Stadt Budapest."

TABLE VI.-Carcinoma of Uterus. Budapest 1902-1906. (Theilhaber, 1910.)

Total.     Jewish.    Jewish               Cancer of uterus

A.          B.      per cent  Ratio B: A   per cent of all

of total.                cancers.

Deaths         . .  71,700  .  12,605  .  17.6   .  1: 5.7   . Total    17.7
Deaths from

cancer  .    4,426  .     883   .  19.9   .  1: 50

Deaths from                                                     Jewish   7.7

cancer ofuterus    783   .      68   .   87    .  1 :.11.5

Presumably the total deaths, and deaths from cancer, are those of women only,
but this is not stated. A rather larger fraction of the total deaths was due to all
forms of cancer among Jews than among the general population, but Jewish
deaths from cancer of the uterus showed a much lower ratio.

Sorsby (1931) also gives data for cancer of the uterus from Vienna and Buda-
pest, which have been criticized by Peller (1931), and for cancer of the whole
female reproductive tract from Warsaw.
Amsterdam.

Hoffman (1933) obtained data, some of which have been put together in
Table VII, from manuscript material at the Municipal Bureau of Statistics in
Amsterdam, for the incidence of cancer in relation to religious confession in that

181

E. L. KENNAWAY

city. The results are given under three headings, namely, Jews, Catholics and a
third referred to as "Protestants" or "others."    The figures show that the
mortality from cancer of the uterus is much lower, and that from cancer of the
ovary, and to a less extent that from cancer of the breast, is higher, in Jews.
Unfortunately the absolute numbers of cases in the three groups are not given.

TABLE VII.-Cancer Mortality of Religious Sects, Amsterdam, 1920-1929.

(Hoffman, 1933.)

Percentage of all cancers in  Rates per 100,000 of both sexes.  Average age at death.

women.

Cancer of-            Cancer of-         Cancer of-

Ovaries.  Uterus.  Vagina. Ovaries.  Uterus. Mamma. Ovaries.  Uterus.
Jews .     . 7-1 .  5-5  . 0 8 . 5-02 . 3-91 . 11-3 . 53-2 . 60-3

Protestants. 2-7  . 139  . 0 9 . 1-96 . 9-78   . 9-65  f541           " all

Catholics  . 2 8 . 14 2 . 0 6 . 2 04 . 10 19 . 932      '              faiths."

Sorsby (1931) gives the following figures (Table VIII) taken from official
publications to which no reference is given.

TABLE VIII.-Cancer of Uterus. Amsterdam, 1922-26. (Sorsby, 1931.)

Rate per 100,000 women.

Number of       '

Number of          ~~~Aged 40 years

deaths.        All.        anged 0 yover.

Non-Jewish women       .    505     .    18.1     .    60.5
Jewish women      .    .     13     .     6 9     .    19.9

One must assume that these data are included in those given above from
Hoffman for a longer period. The low incidence in Jews is the more noteworthy
because they have, in Amsterdam, a somewhat higher proportion of older persons
(Table IX).

TABLE IX.-Amsterdam-Percentage of Persons of 40 Years of Age and Over.

(Sorsby, 1931.)

1909.         1920.

Non-Jews .       .    .    .    27-4     .    28 8
Jews .     .     .    .    .    284      .    32.9

Sorsby (1931) quotes a thesis by J. Sanders which I have been unable to obtain,
who, "speaking of Amsterdam, gives the incidence of cancer of the uterus per
100,000 individuals, 11*3 for Jewesses and 23 for non-Jewesses . . ."
Rotterdam.

J. Sanders (1916) gives data from Rotterdam (Table X) which show a low
incidence of cancer of the uterus in Jews, and a still lower incidence upon members
of two of the Protestant Churches (Christian Reformed and Reformed). He is
unable to account for this difference among Protestants, which is shown also by
figures for other forms of cancer and for tuberculosis, and cannot be explained
by any difference in social conditions.

182

INCIDENCE OF CANCER OF UTERUS

TABLE X.-Deaths According to Religious Confession of Women in an Average

Year of the Period 1902-1914 in Rotterdam per 100,000 Living. (J. Sanders).

Dutch   Roman and  Christian

Reformed. Old Catholic. Reformed.  Lutheran.  Jews.
Cancer.            W. Reformed.        Reformed

Church.

Stomach    .       .    .   28- 8  . 23-1  . 111    .   28 7  .   21.2
Uterus   .    .    .    .   23 8  . 22 8   .   5.2  .   21.1  .    8.2
Breast  .     .    .    .    94   . 10.4   .   4.8  .   15 3  .   14.7
All cancers   .    .    . 122.7   . 97.4   . 34.8   .   109.2 . 106.1

Sweden.

Dr. J. Heyman (1948, private communication) writes: "There are about
seven thousand Jews in Sweden, i.e. about one per thousand of the population.
The Radiumhemmet serves a well-defined area of the country which covers about
half of the population. Since 1914 practically all cases of cancer of the cervix
occurring within that area are referred to the Radiumhemmet for treatment.
From 1914 to 1947 inclusive I have seen about 7000 cases of cervical cancer.
Among those were three Jewish women. The "normal" figure would have been
seven. Consequently the incidence is low. Our figures are, however, much too
small for drawing conclusions."

The following publications, which I have been unable to see, are referred to
by Davidsohn (1939). Auerbach (1908) "found the mortality rate in Budapest
for uterine cancer per 100,000 of the-population to be 8.6 for Jewish women and
25 for non-Jews." Adamowickowa (1929, 1932) published three papers in a
Polish journal on the incidence of cancer on Jewish and other women in Warsaw,
but, as quoted by Davidsohn, he does not distinguish cancers of the uterus from
among all cancers of the female genital organs.

Palestine.

At the Copenhagen Cancer Statistics Conference in 1946, Dr. Karplus, Patho-
logist to the Municipal Hospital, Tel-Aviv, reported that cancer of the uterus
was not common in Jewish women, but that the most striking thing was that the
relation between the incidence of cancer of the body and cancer of the cervix
was reversed in Jews. In his experience in Tel-Aviv cancer of the cervix was
rare and there were four cases of cancer of the body to each one of the cervix.
(At the Royal Cancer Hospital (Free), London, the ratio of cervix to body is
about 6 or 7 to 1). He considered that this was almost certainly an hereditary
factor and not likely to be due to special observances of the Jewish Law, the same
facts being noted in unorthodox Jews.* Unfortunately Dr. Karplus gave no
figures in support of this statement. Data showing an equally low incidence
of cancer of the uterus, which is more difficult to demonstrate than a high inci-
dence, in populations known to consist of orthodox, and of unorthodox Jews must
have been difficult to obtain and would be of great interest.

* I am indebted to Prof. D. W. Smithers for this account of Dr. Karplus' contribution.

183

E. L. KENNAWAY

New York.

Fishberg (1902) gives data obtained (a) from the "Annual Reports" for 1898,
1899, 1900 of a Jewish hospital (Mt. Sinai) for both sexes; and (b) "from a general
hospital which admits few Jews (St. Luke's)," both hospitals being in New York
City (Table XI).

TABLE XI.-Cancer of the Uterus at Mount Sinai and St. Luke's Hospitals,

New York. (Fishberg, 1902).

Mount Sinai (Jewish), 9497 pts.  St. Luke's, 7933 pts.

r     <-------

Parts affected by cancer.  Cancer cases. Percentage of  Cancer cases.  Percentage of

cancer cases.               cancer cases.

Uterus      .    .    .    18     .   7      .0  .    59     . 16.0
Breast      .    .    .    32     . 124          .    91     . 24- 6

Gastro-intestinal tract .  81    . 31-3)         .    48     . 13.0j

Liver .     .    .    .    19     .  7.4   43.4  .    14     .  3 8   23-5
Rectum      .    .    .    12     .  4.7J        .    25     .  67J
Other organs     .    .    38    . 14.7          .    86    . 23.2
Sarcoma     .    .    .    58     . 22.5         .    47    . 12.7

Total   .    .    .   258    . 100           .   370    . 100

Table XI shows that cancer of the uterus, reckoned as a percentage of all
cancers (Fishberg does not make clear whether the figures refer to females only)
is less than one-half as common among Jewish women as among other races,
while cancer of the breast shows a smaller difference in the same direction.

Smith (1941) studied the racial incidence of 3106 cases of cancer of the cervix
observed at the Memorial Hospital, New York, from 1916 to 1937. He says:

"In an attempt to compare the nationality incidence in cervical cancer in
this series with that of patients suffering from other gynecological lesions, con-
secutive records as filed have been studied, but the numbers used in each classifi-
cation are not the entire number of patients filed under that diagnosis; i.e., this
is a cross section of the clinic in the years studied rather than the total number
of cases in the clinic."

I have been unable to learn from the Memorial Hospital the system upon
which the cases making up this "cross section" were selected. Apparently a
number of cases of other gynaecological conditions roughly equal in number (3062)
to those of cancer of the cervix (3106) were taken. The results are summarized
by Smith in Tables XII and XIII (his Tables IV and IVa).

The significance of these figures is affected by the very high proportion of
benign tumours, hence in Table XIV and XIVA Smith's figures have been recal-
culated for the malignant tumours only.

In Table XIV the result of the whole investigation, which for some reason
Smith does not give, namely, the ratio of cervix cancers to other conditions, is
calculated from his figures. The results show that cancers of the cervix make up
58-5 per cent of all the conditions studied in the non-Jewish peoples and only
12.2 per cent in Jews, while if the non-malignant tumours are excluded the respec-
tive percentages are 82.3 and 48-4.

184

INCIDENCE OF CANCER OF UTERUS

TABLE XII.-" Comparison of Percentages of Nationality Incidence in the Several

Types of Gynecologic Lesions." (Smith, 1941.)

Black  .
German
Italian

Jewish .
Greek  .
Irish    .
Polish

English and Scotch
Austrian
U.S.A..
Others .

Total

Cervix.

7'2 .
109 .
16-9 .
4-2 .
0 3 .
13'4 .

10 .
10'6 .
3-3 .
24'5 .

6'7 .

Corpus.

,00

3'1 .
7'6 .
7'1 .
19'6 .
.0'0 ..
186 .
0'4
0'0

206 .
35-2 .
5*6.

Ovary.

1*8 .
5-4 .

4'0.

4 0 .

21*6 .

00 0

3'1 .

0*0

0'0 .

4'9 .
7'2 .
40' 7 .

5'4 .

Vulva.

60/

4'3 .

6'0 .

8-6 .
7'8 .
9'0 .
15'6 .
0'8 .
0'0 .
4'3 .
50'4 .

1'7 .

Benign.

7'8 .
8'1 .
8'6 .
35.5 .

1 4 .
15.8 .

3'0 .
8.6 .
3.2 .
5-6 .
1.0 .

Clinic.

No.    %
426 . 6 9
582 . 9.4
777 . 12 5
1085 . 17 5

43 . 0.06
890 . 14-4
104 . 1.6
439 . 7- 1
219 . 3.5
1191 . 19- 3
412 . 6 6

. 3106 . 445 . 221 . 115 . 2281 . 6168 . --

TABLE XIII.--" Comparison of Nationality Incidence in Patients with Cervical and

Non-cervical Lesions and with a Cross Section of the Entire Clinic." (Smith,
1941).

Black

German
Italian
Jewish
Greek
Irish

Polish

Scotch and English
Austrian    .
U.S.A.
Others

Total .

Cervix.

No.       %
225   .  7 2
341   . 10 9
528   . 16.9
132  .   4-2

10  .   0 3
419   . 13.4

32   .   1.0
330   . 10.6
104  .   3.3
764   . 24.5
221   .  7.1
. 3106 .

Clinic.

-              IA

No.

426
582
777
. 1085

43
890
104
439
219
. 1191

412

6168

6-9
9.4
12.5
17.5

0.06
14-4

1I6
7*1
3.5
19 3
6*6

Non-cervical.
No.      %
201   .   6-5
241   .   7-8
249   .   8 1
953   . 31.1

33   .   10
471   .  15.3

72   .   2*3
109   .   3 5
115   .   3.7
427   .  13 9
191   .   6* 2

3062

Vineberg (1919) gives data for the incidence of cancer of the cervix based upon
material from 80,000 female patients at the Mount Sinai Dispensary and Hospital,
New York. Table XV represents an attempt to reduce his rather confused state-
ment to a clearer form. The higher proportion of cancer in the hospital cases as
against those of the dispensary is attributed to better conditions for examination.

Vineberg (1919) points out that Jewesses of the poorer classes, to which most
of those dealt with in his paper belong, are most likely to be orthodox in religious
matters. "It is well known that the poorer classes of non-Jewish women not only
do not observe such restrictions, but are in the habit of indulging in cohabitation
during the menstrual period and very shortly after parturition."

185

E. L. KENNAWAY

TABLE XIV.-Cases of Carcinema of Cervix as Percentage of all Gynecological

and of all Gynecological Malignant Cases.  Memorial Hospital, New York,
1916-1937. (Smith, 1941.)

Black

German
Italian
Jewish
Greek
Irish .
Polish

English and Scotch
Austrian  .
U.S.A.
Others

A.        B.        C.

Carcinoma All malignant All gyneeo-
of cervix.  tumours. logical cases.

225    .   248    .   426
341    .   395    .   582
528    .  579    .   777
132   .   273    . 1085

10   .    10    .    43
419    .  527    .   890

32    .   35    .   104
330    .  341    .   439
104   .   137    .   219
764    . 1069    . 1191
221    .  260    .   412

Total .

3106

Total non-Jewish .

2974

. 3874
. 3614

. 6168
. 5083

All

A -

79.9       50-4
All non-Jewish
82-3  .    58.5

TABLE XIVA.-Cases of Carcinoma of Corpus as Percentage of all Gynecological

and of all Gynecological Malignant Cases. Memorial Hospital, New York,
1916-1937. (Smith, 1941.)

Black     .
German . .
Italian
Jewish
Greek
Irish .
Polish

English and Scotch
Austrian  .
U.S.A.
Others

A.        B.         C.

Carcinoma All malignant All gynaeco-
of corpus.  tumours. logical cases.
-  14    .   248   .   426

35     .   395    .   582
32     .   579    .   777
85     .   273    -  1085
0      .    10   .    43
83    .    527    .   890

2      .    35    .  104
0      ?   341   .   439
12     .   137    .   219
157    ?   1069   - 1191
25     .   260    .   412

Total .

445

. 3874

. 6168

360   . 3601    . 5083

All
,   -A

11-5   .   7-2
All non-Jewish
10.0   .   7-1

A per cent

of B
90- 7
86-3
91-2
48-4
100

79.5
91-4
96- 8
75.9
71-5
85-0

A per cent

of C.

52-8
.58-6
68-0
12-2
23-3
47-1
30- 8
75-2
47.5
64-2
53-6

A per cent

of B.
5-6
8-9
5.5
31-1

0-0
15-8
5.7
0-0
8-8
14-7

9-6

A per cent

of C.

3.3
6-0
4-1
7-8
0-0
9.3
1-9
0-0
5.5
13-2

6'1

186

Total non-Jewish .

INCIDENCE OF CANCER OF UTERUS

TABLE XV. -Cancer of the Cervix in Jewish and Non-Jewish Women in N6w York.

(Vineberg, 1919.)

Approximate
proportion of

Jewish
patients.

Mount Sinai
Dispensary

1893-1906   . 95%       .

New gynaeco-

logical

patients.

19,800

1909-1918 . 14/15 . ca. 30,000

Mount Sinai
Hospital

1911-1918 .

New female

patients.

14/15 . ca. 30,000*

Cases of

"marked laceration

of cervix."

Cancer of cervix.

Jews.          Non-Jews.

1995    .      9       .      9

(1in 2089)  .  (1 in 111)

13      .       7

(1 in 2154)  . (1 in 285)

. 32 in 28,000

(1 in 875t)

. 33 in 2000

(1 in 61)

* Elsewhere in the same paragraph Vineberg says that these 30,000 cases included" carcinoma
of the cervix 58 cases; of the uterus 35 cases . . . ," but gives no hint of the relation of these
88 cases to the 32 + 33 cases set down in the Table.

t Vineberg states this proportion to be 1 in 973.

Mayo Clinic.

Horwitz (1927) made a study of the records of all cases of primary carcinoma
of the uterus (cervix or fundus) occurring in the Mayo Clinic between 1920 and
1925 inclusive (Table XVI). The proportion of Jews registering at the Mayo
Clinic was estimated, by a method which is not very clearly described, at 5 per
cent.

TABLE XVI.-Analysis of 1,237 Cases of Primary Carcinoma of the Uterus from

1920 to 1925 inclusive at the Mayo Clinic.  (Horwitz, 1927.)

New patients registered at the Mayo Clinic (1920 to 1925 inclusive) .
New Jewish patients (estimated at slightly less than 5 per cent)

Carcinoma of the uterus (Gentiles) (550 for each 100,000 Gentile regis-

trations)    .     .         .     .    .     .    .

Carcinoma of the uterus (Jewesses) (86 for each 100,000 Jewish regis-

trations)    .     ..              .    .     .    .

234,250

11,700

1,227

10

Married women
Single women
Nulliparae .

All women.

Number.     Per cent.

1202    .   97-0

35    .      3' 0
124    .   10'3

Jewesses.

Number.        Fer cent.

10      .     100
0       .        0

1

10

Total   .    .    .    .   1237

Average number of.pregnancies 4*43 in 1078 patients .
Average age of women with car-

cinoma of uterus, years  .       49.93

10

4.67 in 9 patients

45.40

187

188                          E. L. KENNAWAY

Horwitz also gives data from the Curie Hospital attached to the Mayo Clinic
(Table XVII).

TABLE XVII.-Analysis of Cases of Carcinoma at the Curie Hospital from 1920

to 1925 inclusive. (Horwitz, 1927.)

Gentiles.                Jews.

Number.     Per cent.   Number.     Per cent.

Registered from 1920 to 1925

inclusive  .     .    .    .   6630    .    -      .    206    .

Diagnosis of carcinoma  .    .   4402    .    66     .    129    .   62.0

Per cent of             Per cent of
carcinomas.             carcinomas.

Carcinoma of the uterus .    .    727    .   16.5    .      5    .    3.9
Carcinoma of the breast .    .    1246   .   28 3    .     34    .   26-4
Carcinoma of the digestive tract,

including liver and pancreas    753    .   17 1    .     39    .   30 2

Total    .    .     .    .   2726    .   61.9    .    78     .   60.5
Chicago.

Davidsohn (1939) studied the incidence of cancer of the cervix, and of the
corpus, in the Mount Sinai Hospital, Chicago, during the years 1930-38 (Table
XVIII).

TABLE XVIII.-Carcinoma of Body and Cervix of Uterus, Mount Sinai Hospital,

Chicago.  (Davidsohn, 1939.)

1930-1938.                      Total.      Jewish.     Non-Jewish.

Female patients over 14   .     .    . 21,673    .  ca. 90%    .  ca. 10%
Endometria fUteri, 942                   2002
examined      Curettings 1060 f

Cervices f Uteri   942                    17

examined Cervices 136                      08          -             -
Carcinoma of body    .    .    .     .      21   .    15       .      6

Per cent of endometria* .    .     .      -   .     0-83    .       3
Carcinoma of cervix  .    .    .     .      16   .    9        .      7

Per cent of cervices*   .    .     .           .    0-93     .      6.4

* These percentages, given by Davidsohn without explanation, will be found to imply that the
ratio-90 Jewish to 10 non-Jewish-shown by the hospital population applies also to both parts of
the uterus examined, which is, of course, not necessarily the case.

New York, Chicago and Philadelphia.

Weir and Little (1934) collected data from 18 general hospitals in Chicago,
New York and Philadelphia showing the distribution of "cancer of the cervix
and uterus" among Jewish and other women. They give a table, which is given
in an abbreviated form in Table XIX, showing "Comparison of Jews among
cases of cancer of the cervix and uterus with total admissions in eighteen general
hospitals."  Presumably the "total admissions" are those of women only.

INCIDENCE OF CANCER OF UTERUS

TABLE XIX.-" Comparison of Jews among Cases of Cancer of the Cervix and

Uterus with Total Admissions in Eighteen Goneral Hospitals."  Weir and
Little (1934).

Patients 1927-1931 inclusive.          Cancer cervix and uterus.

A ^            M         .             A

Total.      Jewish.      Per cent       Total.      Jewish     Percent

Per cent                    ~~~~~~~~Per cent
Jewish.        T                      Jewish.

572,232  .  111,447   .    19.48    .    1842     .    126    . 6 84

Weir and Little give another table of data from five Jewish hospitals in New
York, Brooklyn, Cleveland and Philadelphia (Table XX).

TABLE XX.-" Comparison of Jews among Cases of Cancer of the Breast and

Cancer of the Cervix and Uterus in Five Jewish Hospitals." Weir and Little
(1934).

Cancer of oervix and uterus.             Cancer of breast.

Total cases. Jewish cases. Per cent Jewish.  Total cases. Jewish cases.  Per cent Jewish

199    .    72     .   36.18   .    448     .    310    .    69.2

Unfortunately the authors speak only of "cancer of the cervix and uterus."
I have been unable to ascertain whether figures for cancer of the cervix and of
the corpus uteri separately are available.

Weir and Little (1934) give a graph (Fig. 1) showing the percentage age dis-
tribution in quinquennial periods of 598 cases of "uterine cancer" in Jewish
and other women from eight hospitals in New York, Philadelphia, Cleveland and
Chicago. The maximum is in the age group 50-54 in Jewish women and in that
of 45-49 in others. The authors suggest the possibility that this difference
depends upon racial factors. Certainly the age at which some forms of cancer
occur is affected by genetic differences; examples have been given in detail in
another paper (Kennaway and Kennaway, 1944). But there is nothing in
these data for age distribution to indicate whether the liability to uterine
cancer, which is the chief subject considered here, is affected by racial or by
extrinsic factors. A larger proportion of cancers of the corpus uteri among
Jewish women would have a similar effect (cf. Karplus p. 183; "Genetic
Factors" and" Cancer of the Corpus Uteri," pp. 204, 205, below).

Summary of data on cancer of the uterus in Jewish women.

Table XXI brings together the data of different authors, which are expressed
in a variety of ways, and cannot be referred to any one base-line; some are
derived from the statistics-of whole countries and cities, others from admissions
to one or more hospitals.

The figures for cancer of the uterus, reckoned as a percentage of all cancers
in women, range in the first seven cases in Table XXI from about 28 to 14 in
non-Jews, and from 10 to 4 in Jews, the mean values being about 20 and 7. A
similar ratio of the order of 3 1 is given by the death rates per 100,000 of Hoff-
man (Amsterdam), Sorsby (Amsterdam), Sanders (Rotterdam), Auerbach (Buda-
pest); and by the figures of Davidsohn (Chicago) for cancer of the corpus uteri.

189

E. L. KENNAWAY

I 'S

P-

Age group in years

FIG. 1.-Age distribution of uterine cancer (Weir and Little, 1934).  Gentiles, - - - - Jews.

A smaller difference, of the order of 2: 1 or less is shown by the data of Sanders
(Amsterdam), Peller (Vienna), and by the hospital admissions for malignant
tumours in women of Smith (New York). Other results from hospitals (Horwitz,
Mayo Clinic; Davidsohn (cervix) and Vineberg) show much larger differences, up
to 19 :1. The difficulty of comparisons based upon admissions to hospitals is
of course well known. Three items in Table XXI (F. Theilhaber, 1910; A.
Theilhaber, 1909; Weir and Little, 1934) are not comparable with the rest.
But allthe 20 sets of data in Table XXI agree in attributing to Jewish women an
incidence of cancer of the uterus lower than in other women, with the single
exception of those of certain Dutch Reformed sects (Table X).

B. Cancer of the Uterus in Hindu, Moslem, Parsee and Indian Christian Women.

Statistics based upon attendance at hospitals are notoriously liable to error,
more especially when they refer to people differing in race and religion. However,
the following figures (Table XXI1) should at any rate encourage further investi-
gation. They are taken from the valuable collection of data on cancer in India
obtained by Nath and Grewal (1935) from the records of in-patients of 13 hos-
pitals in Punjab, Delhi, United Provinces, and Bihar and Orissa, comprising
6395 cases of cancer in all. The figures as they stand show a lower proportion

190

I

INCIDENCE OF CANCER OF UTERUS

' 191

TABLE XXI.-Cancer of the Uterus in Jewi8sh Women. Summary.

Locality.
Munich

1876-1908
Munich

London

1911-1920

Budapest
1902-1906
. Amsterdam
? 1920-1929

New York
. 1898-1900

? Curie Hospital

1920-1925
Budapest
1901-1905
. Amsterdam

Rotterdam
1902-1914

? Amsterdam

? Amsterdam

1922-1929
-    Vienna

1926-1929
Mayo Clinic
1920-1925
New York
1916-1937

Chicago

1930-1938

Vineberg . New York

(1919)      Mt. Sinai

Dispensary

Theilhaber .

(1910)

Theilhaber

(1909)

Weir and .

Little
(1934)

Mt. Sinai
Hospital

Budapest
1902-1906
Munich

New York,
Chicago and
Philadelphia

Cancer of uterus.

Per cent of all cancers in females

.?~ -        Ditto

Ditto England and Wales, whole popu-

lation
,,  London

? ,, total population (? female)

?  ,,  Protestants

Catholics

,,    (? female) St. Luke's Hospital

Mt. Sinai Hospital

1 .   ..

Death rate per 100,000 both sexes

Ditto Protestants

,, Catholics

Death rate per 100,000 women:

Reformed

R. Catholic
Ditto

Death rate per 100,000 women
Ditto aged 40 and over

Per cent of all cancers of female genital

tract

Cases per 100,000 registrations

(?sex)

Per cent of all gynaecological cases

Per cent of all malignant gynaeoological

tumnours

Carcinoma of cervix per cent of cer-

vices examinec

Carcinoma of body per cent of endo-

metria examined

Ratio to admissions

Ditto

Jewish per cent of all cancers (? females)
Jewish per cent of all cancers of uterus
Ratio uterine myoma: cancer of cervix

Per cent of admissions

Per cent of "cancer of cervix and

uterus"

Non-Jews.       Jews.

25-35     .       7

28-7     .     6-8

(1907-9)   .  (1880-1908)

17-9     .      -
?  -~-  .  10.5

17.7     .     6-7

13-9     .     5-5
14-2     .     -
16.0            -

7 -0
?_-         .      7.0

16.5      .     3.9
25      .     8-6

9-78     .     3-91
10-19

23 -8          8-2
-     22 '8

23-0       .     11-3

18-1       .      6-9
60-5       .     19.9
76        .      48
550        .      86

58-5       .     12-2
82-3       .     48-4

6-4     .      0 -93
3 -0      .      0-83

? 1893-1906

1 in 111      1 in 2089

(0-9 per cent) .(0-048 per cent)

1909-1918

1 in 285      1 in 2154

(0-35 per cent) (0 046 per cent)

1911-1918

1 in 61       1 in 875

(1 - 64 per cent) (0 - 114 per cent)
?  --     -~  .   19.-9
?-~  -         .8-7

1-7 :1     .     43:1
(all patients)

19.-5

6-8

Author.

Theilhaber

(1910)

Theilhaber
& Greischer

(1910)
Sorsby
(1931)

Theilhaber

(1910)

Hoffman

(1933)

Fishberg

(1902)

Horwitz

(1927)

Auerbach

(1908)

Hoffman

(1933)

Sanders

(1916)

Sanders

(1916)
Sorsby
(1931)
Peller
(1931)

Horwitz

(1927)
Smith
(1941)

Davidsohn

(1939)

E. L. KENNAWAY

TABLE XXII.-Carcinoma in Hindu and Moslem Women. (Nath and Grewal,

1935.)

Cases of oarcinoma in women.

'^~ l?~ ~Ratio
A.           B.           C.          AtoC.
Genital tract.  Breast.    All sites.

Hindu    .    .    .    888     .    321    .    1830     .    1: 2-06
Moslem   .    .    .    223     .    102    .     666     .    1: 30

of uterine cancer in Moslem women, which might, of course, be due to various
causes other than a true difference in incidence.

These results of Nath and Grewal are confirmed by the later and much more
detailed observations of Khanolkar (personal communication) on the first 10,000
cases at the Tata Memorial Hospital, Bombay (Table XXIII), which, when
calculated as percentages, show again an incidence of carcinoma of the cervix
which is higher, in proportion to other forms of cancer, in Hindus (44 per cent)
than in Moslem women (20.7 per cent). The Gujarati Hindus, who come from
the coast to the north of Bombay, "form the bulk of the commercial com-
munity," while the Deccanis, from the south and east, "supply the industrial
workers and the lower paid members of the clerical and teaching professions."
Cancer of the cervix"   . . . is most frequently met with in Deccani Hindu
women. A badly balanced, deficient diet, during the child-bearing age may be
a decisive factor in a population generally poorer than the rest." The figures in
Table XXIII show a proportion of cervix cancer which is rather higher in the
Deccani than in the Gujarati Hindus.

TABLE XXIII.-Carcinoma in Womnen at the Tata Memorial Hospital, Bombay.

(Khanolkar.)

Per cent of total

Total    Total  Carcinoma Carcinoma  carcinomas in women.

female carcinomas  of     of             A-        ?

patients. in women. cervix.  breast.  Carcinoma of  Carcinoma

cervix.    of breast.

fDeccani . 1180     . 699   . 329   . 98    . 47         . 14

Hindus . Gujarati .   414  . 230    .   89  . 43   . 38.7   44.0 . 18-7

Others    .   334  . 198    .  77   . 37   . 38.9J       . 18.7
Moslems      .    .   495   . 275   .   57  . 65   . 20.7        . 23.6
Parsees      .    .   478  . 169    .   25  . 83   . 148         . 49

Indian Christians  .  543  . 224    .   69  . 61   . 12.7        . 27.2
Others   .    .   .   269  .   99   .    9  . 41   .   -         .   -

Totals   .    . 3713   . 1894  . 655    . 428

In view of the possibility that Moslem women might avoid the Tata Memorial
Hospital, which has a male medical staff, Khanolkar obtained data also from the
Cama and Albless Hospital for Women, which is staffed entirely by women and is
favoured by Moslem women. The figures (Table XXIV), reckoned on atten-
dances and not on total carcinomas, as in Table XXIII, confirm those from the
Tata Hospital.

192

INCIDENCE OF CANCER OF UTERUS

TABLE XXIV.-Carcinoma of the Cervix in Hindus and Mloslems. (Khanolkar.)

Tata Memorial Hospital.  Cama and Albless Hospital.

Hindus.                Hindus.

Deccani. Gujarati. MIoslems. Deccani. Gujarati. Moslems.

Carcinoma of cervix per cent

of total attendances .   . 278 . 21.5   . 11.5  . 12.5 . 10.1  . 4.8

Ratio. Deccani- 1.0.       . 1.0 . 0.78 . 0-41 . 1.0 . 0.81 . 0.39

The Indian Christians are descended from converts made by the Portuguese
fonr centuries ago. "They are usually very devout Roman Catholic Christians
and circumcision is not practised amongst them. There must have been a good
deal of intermixture of Portuguese blood in the early days." Data from a Jewish
population inhabiting the same area would be of great interest.

The Parsees show a percentage of uterine cancer (14-8) which is even lower
than that seen in Moslems, and among the Indian Christians the percentage is
lower still (12.7). Thus in India, the Hindu women on the one hand, and the
Moslems, Parsees, and Christians on the other, appear, in so far as the very
scanty data allow of any statement, to present a contrast similar to that between
Gentiles and Jewesses in other parts of the world.

c. Cancer of the Uterus in China.

Maxwell (1929) in his book "The Diseases of China" says: " It is probable that
uterine cancer ranks highest of all in the malignant growths in this country. Nor
is this surprising considering how high the birth rate in the country districts is,
or on the other hand, how frequent gonorrhoeal affection is in the cities and what
a lack in both cases there is of cleanliness. In my own experience cancer of the
cervix occurs in comparatively young people, it has often been seen in the thirties
and in two cases in women between twenty and thirty. One of the most advanced
cases of cancer of the cervix which I have seen was in a woman of twenty-three."

SOCIAL INCIDENCE.

The first to draw attention to the social distribution of cancer of the uterus
appears to be A. Theilhaber, a gynaecologist of Munich, and his paper (1909) is
perhaps the first to deal with the social, as distinct from the occupational, incidence
of cancer on any one organ.
Bavaria.

Theilhaber (1909) records 133 cases of carcinoma of the cervix and 228 of
mnyoma of the uterus among 5848 female patients treated by himself; cases of
carcinoma of the body are excluded (Table IV). All the 361 cases are classified
according to the occupation of the husband; nothing is said of the exclusion of
any single women from the series. The numbers attending the practice of a
single surgeon do not provide any basis for generalization and the age distribution
among the various classes is unknown, but Table IV shows that the three highest
social classes gave 35 per cent of the cases of fibroids and only 3.7 per cent of those
of carcinoma of the cervix.

14

193

E. L. KENNAWAY

Theilhaber and Greischer (1910) give a classification of occupations in four
groups, A to D, which is reproduced in Table XXV. Presumably Groups A to
D referred to in other papers by A. Theilhaber (1909) and F. Theilhaber (1910)
have the same significance.

TABLE XXV.-Social Classes. (Theilhaber and Greischer, 1910.)

Group A: Physicians, surgeons, lawyers, high officials, manufacturers, mer-

chants, heads of firms, dentists, architects, persons of inde-
pendent means.

Group B   Innkeepers, butchers, officials of middle rank, teachers, commer-

cial professions not under A.

Group C: Independent manual workers, master craftsmen.

Group D: Domestic servants, journeymen, skilled craftsmen, bricklayers,

mechanics, machinists, day labourers, unskilled workers,
agricultural labourers, lower officials, pensioners, paupers,
sempstresses, laundresses, factory hands.

Theilhaber and Greischer proceed to give the data shown in Table XXVI
derived from the death certificates of women of ages over 25 in Munich for the
years 1907-8-9; The proportion of deaths due to cancer of the uterus rises with
descent in the social scale, while cancer of the mamma varies in the opposite
direction, but in both series Group B shows an especially high incidence. There is
no increase in passing from Group C (24.3) to Group D (24.6), but then the social
difference between these two groups (Table XXV) is perhaps not very great.

TABLE XXVI. Deaths of Women, Munich, 1907-8-9. (Theilhaber and Greischer,

1910.)

A.     B. Deaths from cancer of uterus. Deaths from cancer of mamma.

Deaths

Social class  Total  from   Total. Per cent Per cent  Total. Per cent. Per cent
of husband.  deaths. cancer.        of A.   of B.         of A.   of B.

Group A     .  560     77   . 12     2.1    15.5  .   9    1.6    11.6

,,  B  .   302     32  . 13     4.3    40.6   .  6    1.9     18.7
,,  C  .   594     74  . 18     3      24.3   .  7    1.1     9.5
,,  D  .1373      239  . 69     5      246 .    19    1.3     7.9
Total  . 2829     422   . 112   3.96    26.5  . 41     1- 45   9.7

F. Theilhaber (1910) followed the indications of the study by A. Theilhaber
(1909) of his own cases by a wider investigation of the death certificates of Bavaria
for 1908, which yielded 667 undoubted cases of uterine cancer. He proceeds to give
a table (p. 480 of his paper) containing 863 cases, but does not comment upon the
difference in these totals. The women are classified according to the husbands'
occupation into four main classes, with 14 numbered and 35 named sub-divisions.
In Table XXVII, only the four main classes are quoted.

Theilhaber does not state the populations of these various groups, and makes
no comment upon this very serious deficiency. Figures for the age distribution
of the populations of these classes were not available. The detailed table shows
that, in 1908, in Bavaria apart from Munich, only a single case occurred among

194

INCIDENCE OF CANCER OF UTERUS

TABLE XXVII.-Deaths from Cancer of Uterus, Bavaria, 1908. (F. Theilhaber.)

Deaths.

Social class of husband.

Number. .   Per cent of

total.

Group A: Well-to-do-classes (wohlhabenden), indepen-

dent, professional, commercial  .   .      62    .     7
,,  B: Middle classes  .    .    .     .       .     60     .     7
,,  C: Farmers, manual workers .    .    .     .    114    .     13
,,  D: Labourers    .    .     .    .    .          627     .    73

863     .   100

the wives of ministers of religion, medical practitioners, lawyers, university
teachers, engineers, merchants and manufacturers, while there was a high inci-
dence upon the wives of inn-keepers and butchers.

Theilhaber then obtained the figures for deaths from cancer of the uterus from
Munich only for the years 1906-1907, which were in Group A, 22, Group B, 22,
Group C, 24, Group D, 135 (for Groups see Table XXV). In the absence of
figures for the corresponding populations he took the numbers of deaths from all
causes of women 24 years old (presumably he means" 24 years old and upwards ")
for a few occupations only, for 1906 only, and by this not very laborious method
obtained a result indicating an incidence of cancer of the uterus higher in Group B
than in Group A. It seems impossible to reconcile some of his statements with
some of his figures, but Table XXVIII is an attempt to reproduce his results.

TABLE XXVIII.-Deaths from Cancer of Uterus, Munich, 1906. (F. Theilhaber.)

Number of deaths.    Cancer of

Cancer of

uterus per cent
Occupation of husband.           Women-all    Cancer of  Of all deaths.

causes.     uterus   of all deaths.

Higher officials, university posts, artists,

authors, merchants, manufacturers,

shopkeepers, teachers   .    .    .    273     .     3     .    1 1
Middle rank officials, butchers, inn-keepers  172  .     12    .    7 0

Theilhaber gives, finally, in a rather confusing form,-data for the age at death
from cancer of the uterus in the richer and poorer classes of Bavaria. He does
not always make clear the occupations included in the two catergories of "Wohl-
habende" and "Arme," but Fig. 2 and Tables XXIX        and XXX have been
prepared from the material, and these show the earlier age at death of the poorer
women.

TABLE XXIX.-Average Age at Death from Cancer of the Uterus, Bavaria. 1908.

(F. Theilhaber, 1910.)

Social class                   Munich.    Five other large  Province of
of husband.                       ic.     Bavarian towns. Unterfranken.

Group A: Well-to-do     .    .    .    54-9     .    57. 7    .    581

,,  B: Middle class .   .     .    538      .     -

,,  C: Manual workers      . .     530      .     -      .     -
,,  D: Labourers   .       . .     52- 2    .    48 9    .    51- 4

195

E. L. KENNAWAY

TABLE XXX.-Aye at Death from Cancer of Uterus in Bamberg, Augsburg, Wiurz-

burg, Erlangen, Niirnberg and the Province of Unterfranken (1908) and in
Munich (1906-7-8). (F. Theilhaber, 1910).

Age.     Wohlhabende.  Arme.
20-30    .    -     .     9
31-40    .     3    .    46
41-50    .     9     .   80
51-60    .    11    .    79
61-70    .    16    .    51
71-90    .     2    .    13

41    .   278

Kauffmann (1926) in a lengthy study of 2000 cases at the Frauenklinik,
Berlin, in the years 1912-1923, gives a table (p. 212) showing that 77 per cent
of cervix cancers occurred in poor, and 23-3 per cent in rich, women, but he does
not give the proportions of these two classes which attended the clinic.
England and Wales.

Data for the social distribution of cancer of the uterus (corpus and cervix),
were given by the Registrar-General (1938) for the first time in the Decennial
Supplement based on the census of 1931. This study was an expansion of that
devoted to the social incidence of other forms of cancer in the preceding Decennial
Supplement (1927) dealing with the 1921 census. In the publications of the
Registrar-General (1927, 1938) "each census unit of occupation has been assigned
to one of five graded Social Classes . . . after consultation with the Ministry
of Labour. So far as is possible from the material available, Class I purports
to represent the professional and generally well-to-do section of the population,
Class III, skilled artisans and analogous workers, and Class V, labourers and other
unskilled callings, while Classes II and IV are intermediate, comprising occupations
of mixed types, or types not easily assignable to the classes on either side."
Married women are classified according to the occupation of the husband as
stated on the death certificate. "Only about 10 per cent of married women were
recorded as gainfully occupied at the census of 1931, and of these about one third
were employed in textiles and dressmaking, and about one third in domestic and
personal service" (1938). Hence the mortality of the great majority of married
women would show the effect of the environment to which they, and their hus-
bands, are exposed, without that of any special occupational factors. (For a
discussion of this matter see p. 3 et seq., Registrar-General, 1938.)

The social class of single women is that indicated by their own occupation.
The significance of the figures for married womenf has been discussed by'Suther-
land (1947).

The figures in Table XXXI are based upon 7831 cases of cancer of the uterus
in married women, and 1294 cases in single women, in the vears 1930-32. The
table shows that the difference in mortality according to social class is consider-
able at every age both in married and in single women. Thus at ages 35-65 the
rate in married women of Class V is twice that of Class I, and single women in
Classes IV-V show a mortality 44 per cent greater than that of Classes I and 11.

196

INCIDENCE OF CANCER OF UTERUS

TABLE XXXI.-Cancer of the Uterus. England and Wales, 1930-1932.

(Registrar-General's Statistical Review, 1936, text, p. 90.)

Per 1000 deaths
Mean annual death rate  Standardized mortality ratio  from cancer of

per million married  (registered per cent of cal-  all sites at ager
Social class.       women at ages:   culated deaths) at 35-65.  65 and over.

A           __    ,  _

35-     45-     55-65    Married.  Single.  Married. Single.

Class I     .   119  . 264     469 .        65      88        78     62

,, 1I     .   144  . 348.     515    .    78            .   96.

,,1II    .   197   .438 .635        .    99.    110    .   102  .76

,,IV     .   239  .466 .      627       106 .   127        .10    95
,,  V     .   294  . 591    . 754    .   130  . 1           110

All    .   209  . 445    . 519    .   100  .         .   103  .

The gradient of fertility shows a somewhat similar relation to social class. Thus
in 1921 the ratio of registered to 100 calculated births for married males in the
five social classes was 85, 85, 97, 109, 128 and the social distribution of uterine
cancer in married women might be thought to be due to the difference in fertility.
But the data for single women show that the matter is not so simple. "There
must exist factors closely bound up with the social class differentiation, either
selective or environmental or both, which are productive of uterine cancer quite
apart from the parturient histories of the women concerned" (Registrar-General,
1938, p. 48).

United States.

Smith (1941) says that in New York City" Cancer of the cervix is found almost
exclusively in the ward or clinic type of patient, rarely being seen in the type of
patient who can afford private care."
France.

Dr. Denoix of the Ministere de la Sante Publique has been so kind as to inform
me that no data are available of the professional and social incidence of cancer
of the uterus in France.

FACTORS WHICH MAY AFFECT THE INCIDENCE OF CANCER OF THE UTERUS.

(1) Ritual Observances and Ablutions Associated with Menstruation and Child-birth.
(a) Jewish ritual.

The earliest Jewish law prohibits intercourse during menstruation or any
other discharge of blood from the uterus, and for a period of seven days after the
cessation of any abnormal flow. The general prohibition is expressed as follows:

Leviticus, xviii, 19: " And thou shalt not approach unto a woman to uncover
her nakedness as long as she is impure by her uncleanness."

Leviticus, xx, 18: "And if a man shall lie with a woman having her sickness,
and shall uncover her nakedness; he hath made naked her fountain, and she
hath uncovered the fountain of her blood; and both of them shall be cut off
from among their people."

197

E. L. KENNAWAY

The precautions to be taken are stated in an earlier chapter, which makes
plain the abhorrence, which is, of course, by no means restricted to Jews,* with
which any contamination with genital blood was regarded.

Leviticus, xv, 19-28.-" 19: And if a woman have an issue, and her issue in her
flesh be blood, she shall be in her impurity seven days: and whosoever toucheth
her shall be unclean until the even. 20: And everything that she lieth upon
in her impurity shall be unclean; every thing also that she sitteth upon shall be
unclean. 21: And whosoever toucheth her bed shall wash his clothes, and
bathe himself in water, and be unclean until the even. 22: And whosoever
toucheth any thing that she sitteth upon shall wash his clothes, and bathe himself
in water, and be unclean until the even. 23: And if it be on the bed, or on any
thing whereon she sitteth, when he toucheth it, he shall be unclean until the
even. 24: And if any man lie with her, and her impurity be upon him, he shall
be unclean seven days: and every bed whereon he lieth shall be unclean. 25:
And if a woman have an issue of her blood many days not in the time of her
impurity, or if she have an issue beyond the time of her impurity: all the days
of the issue of her uncleanness she shall be as in the days of her impurity: she
is unclean. 26: Every bed whereon she lieth all the days of her issue shall be
unto her as the bed of her impurity: and every thing-whereon she sitteth shall
be unclean, as the uncleanness of her impurity. 27: And whosoever toucheth
those things shall be unclean, and shall wash his clothes, and bathe himself in
water, and be unclean until the even.   28: But if she be cleansed of her issue,
then she shall number to herself seven days, and after that she shall be
clean."

The law thus draws a distinction between normal menstruation (verses 19-24)
and any other genital discharge of blood (verses 25-28). The normally menstruat-
ing woman ceases to be impure, if the flow has ceased, at the end of the seventh
day from its commencement, but after any abnormal flow, a period of seven blood-
free days is ordained, no doubt because there is greater danger of the recrudes-
cence of any such bleeding. The law does not prescribe specifically for women
any purification by washing such as is imposed upon men who have become
contaminated. But subsequently, in Talmudic timest (about the third century
A.D.), the law in regard.to normal menstruation was altered in two respects, and
these changes were set forth about 1000 years later in the Code Yoreh Deah: (1)
The extension of the period of impurity until seven blood-free days had elapsed
was applied to normal menstruation also. (2) The minimum first period, includ-
ing the time of the actual flow, ivas laid down as five days. Hence the whole
period of impurity is n + 7 days, and n is never less than 5, and under normal
conditions will be 5. Such a period of 12 days is nearly one-half of the normal

* The supposed harmful influence of the menstruating woman upon men and children, and upon
a great variety of objects (food and drink of every kind, cooking utensils, the domestic hearth,
weapons, mirrors, crops, trees, livestock, and even upon roads and fisheries) is or has been the subjeot
of stringent precautions and prohibitions among peoples all over the world, from Eskimos to Poly-
nesians (for the immense literature on this subject see Ploss, Bartels and Bartels, 1927). Hence
there is nothing in principle peculiar to the Jewish people in the ordinances of Leviticus, but they are
characterized, as are other parts of the Mosaic law, by great precision in detail, and have received
the voluminous addition of Talmudic literature.

t A tractate (Nilddah) of the Talmud, consisting of 10 chapters, deals with this subject. An
English translation of Niddah is available (Epstein, 1948). No translation of Yoreh Deah into a
European language has been made; the relevant portions of it (chapters 183-200) have been sum-
marized by Sorsby (1931).

198

INCIDENCE OF CANCER OF UTERUS

menstrual cycle and will allow intercourse to be resumed near what is thought
to be the usual time of ovulation, of which process those who laid down the rule
of course knew nothing.

The modern practice follows these rules.  "The law of Niddah is in full force
to the present day" (Epstein, 1948). The precautionary period of impurity
begins 12 to 24 hours before the expected flow, and lasts for not less than 5 days
from its commencement. The woman then examines herself and, if the flow
has ceased, takes a bath. The second period, of seven days, during which she
examines herself repeatedly, follows. If no blood has been found during this
time, she takes another bath, followed by ritual immersion (Mikveh). The test
for cessation of flow is made by inspection of a white cloth which has been
pressed into the vulva.
Ritual immersion.

The Talmudists developed the Mosaic law of Purification by ordaining that a
woman after menstruation should wash her body, and then immerse herself com-
pletely (two or three times) either in a lake, river, or spring, or in a tank containing
not less than the equivalent of 24 cubic feet of water, which must either come imme-
diately from the earth as a spring, or be collected rain water. In medieval times
one of the first cares of every Jewish community was to provide a suitable bath
for this immersion. In these buildings a staircase leads to a dressing-room and
then on beneath the water to the floor of the bath, which was generally below
ground-level so that the ground water, which could be regarded as spring water,
could be utilized. Figures and architectural details of such baths are given by
Ploss, Bartels and Bartels (1927, figs. 460-463) and in the Jewish Encyclopedia
(1891) (article "Andernach ").*  A suitable bath can be constructed in a private
house (Miller, 1930).

Mere immersion in spring water, as an addition to an ordinary bath, could
make little contribution to cleanliness and was not intended to do so; hence, from
the present point of view this particular ritual is in itself of little importance:
but its persistence affords valuable evidence of an obedience to the law which
probably would extend to other ordinances, and especially to the abstention
from sexual intercourse. The object of the preliminary bath was not cleanliness
per se, but the removal of anything which would intervene between the skin and
the water during the total immersion, and thus deprive this ritual of its efficacy.t
Some medical writers have emphasized, in rather vague terms, the importance
of the cleanliness enforced by the Mosaic law, hence it is important to note exactly
what the law requires. The washing, in a normal woman, at the beginning and
end of the seven days, adds two baths a month to whatever other ablutions she
may carry out.

* This account of Jewish ritual immersion is taken from various articles in the Jewish Encyclopedia
(1891), from Ploss, Bartels and Bartels (1927) and from Preuss (1923). The Talmudic ordinances do
not, of course, imply that women had not in earlier times purified themselves after menstruation
by washing (see, for instance, the story of Bath-sheba, Sam. ii, xI. 2-4).

t Chapter X of the tractate Niddah deals with numerous possible obstacles to the access of water
to every part of the body. Thus the woman must not perform immersion in a harbour, because the
passage of ships may stii up mud which would separate the skin from the water, and she must not
raise her eyebrows unduly lest a wrinkle be produced into which the water does not penetrate. The
ritual nature of the immersion is shown by the fact that, before it, all particles of food must be
removed from between the teeth; although the mouth remains closed, there must be no potential
obstacles to penetration. Such regulations are set forth in more accessible publications (e.g.
Hurwitz, 1921) for use at the present day.

199

E. L. KENNAWAY

We have no data to show what proportion of Jewish women in any community
carry out the whole procedure. One might form some estimate in London from
the number of public ritual baths available, which are said to be not more than
half a dozen; there are also some private ones. A new bath for this purpose was
constructed in North London recently. But it seems probable that many women
who do not go to the ritual bath may conform to the law in other respects,
namely, the first and second bath, and abstention from intercourse, which are
matters of purely private conduct.

The Mosaic law (Leviticus xii, 2, 5) relating to child-birth is important in
view of the association of cancer of the cervix with parity. "If a woman conceive
seed, and bear a man child, then she shall be unclean seven days; as in the days
of the impurity of her sickness shall she be unclean. . . . And she shall
continue in the blood of her purifying three and thirty days  . . . But if
she bear a maid child, then shall she be unclean two weeks as in her impurity:
and she shall continue in the blood of her impurity three score and six days."
Thus the whole period of impurity was either 7 + 33 = 40, or 14 + 66 = 80
days. The practice in this country at the present time is said to be, to observe
rather longer periods, of two and three months respectively.

(b) Moslem law.

The Koran (Sura 2) gives directions for the purification of women which are
much less precise than those of the Jewish law. "They will ask thee also con-
cerning the courses of women: Answer, they are a pollution: therefore separate
yourselves from women in their courses, and go not near them, until they be
cleansed. But when they are cleansed, go in unto them as God has commanded
you." No periods of time are laid down, and no details of the method of washing
are given. Obviously, immersion on the Jewish scale would be difficult in
countries where water is scarce. Sale (1861) in his commentary on the Koran,
speaks of two degrees of purification from various contaminations, namely,
immersion or bathing in water, and washing of the face, hands and feet in water
or (Sura 4 and 5) in sand if water is not available; and he goes on to say that the
former is incumbent upon women after menstruation, but this is not in the text
of the Koran.

(c) Parsee ritual.

The Zend-Avesta (Darmesteter, 1880) enacts that a woman having any normal
or abnormal discharge of blood must be placed in a separate building [as is done in
many parts of the world (footnote, p. 198)] and must be prevented as far as possible
from defiling the elements of earth, water and fire; she must be given very scanty
food lest the evil power (Ahriman) within her be strengthened, and the person
who brings this food must not approach within three paces of her, and must use
a metal spoon to convey the food. " If she still see blood after three nights have
passed, she shall sit in the place of infirmity until four nights have passed," and
so on up to a flow lasting 8 nights, one day being added to the actual period.
Then "they shall dig three holes in the earth" (magas) "and they shall wash
the woman with gomez" (urine of cattle) "by two of those holes and with water
by the third." Intercourse with a woman having a normal or abnormal issue

200

INCIDENCE OF CANCER OF UTERUS

of blood is a crime punishable with 200 stripes. A flow of blood lasting more
than nine nights is the work of evil spirits.

Dr. Modi (1922), a Parsee, quotes these laws and says: "At present also,
most of the Parsee women generally observe the above practices. There are no
separate Dastanistans or houses for menses in Parsee towns or streets, but gene-
rally a sequestered part of one's own house is chosen for the purpose. The down-
floor of the house was thought to be the proper place. But nowadays, in a
crowded city like Bombay, the down-floor, instead of being a quiet and healthy
place such as that contemplated by the early injunctions of the Vendidad, is
generally quite the contrary. So, most women in menses pass the period of
menstruation on their upper floors, but in an isolated way. Every family has
a separate iron cot for the occasion and a separate bedding, etc. They are
supplied their meals from a distance by others and they neither come into contact
with others, nor do they touch other things or do household work. The very rigorous
isolation enjoined by the later books is not observed, but anyhow, some kind of
isolation and separation is maintained by the generality of women. In the matter
of taking food, very few use spoons now, though up to about 25 years ago, that
was generally the case. In the matter of purification, they observe the bath
enjoined by the early books, but the Vendidad injunction of bathing over the
three "magas " is not observed at all. A separate place of bathing and for
purposes of nature for women in this condition is generally provided in Parsee
houses."

(d) Hindu ritual.

Dr. Khanolkar (personal communication) says that the Parsee laws regarding
menstruation are almost identical with those practised by orthodox Hindus and
probably both of them are derived from early Aryan ritual. The Abbe Dubois
(Beauchamp, 1906), writing about the beginning of the nineteenth century, quotes
from the book Padma-purama, reputed to be the work of the hermit Vasishta, a
rule for a 3-day p3riod of isolation for the menstruating woman, followed by a day
of ceremonies and ablutions, including 36 total immersions in a river. During the
three days" . . . the mere wish to cohabit with her husband would be a
serious sin." Elsewhere he says, "The mother of the newly-born child lives
entirely apart for a whole month or more, during which time she may touch
neither the vessels nor the furniture of the house, nor any clothes, and still less
any person whatsoever. The time of her seclusion being over, she is immersed
in a bath, or else a great quantity of water is poured over her head and body.
Women are similarly isolated during the time of their periodical uncleanness.
In all decent houses there is a sort of small gynaeceum set apart for them; but
amongst the poor, in whose huts there is no such accommodation, the women
are turned into the street, under a sort of shed or outhouse, or else they are
allowed a corner of the cowshed. . . . When the time of uncleanness is
passed, all the garments that the woman has worn are given to the washerwoman.
Her clothes are not allowed inside the house; in fact, no one would even dare to
look on them."

Jhaveri (1910) says that in all Hindu religious books minute directions for
baths on very many occasions are given. During the first four days of the first
menstrual period everything which the girl touches must be washed and"  . .

201

E. L. KENNAWAY

persons coming into contact with her have to take a bath." This author says
nothing of subsequent periods. After childbirth a woman is impure for 10 days,
even if given a daily bath by the midwife;"  . . . no one dares to touch
her."; after 40 days she is given a final bath, and is then pure (cf. the Jewish
practice after the birth of a male child).

Ploss, Bartels and Bartels (1927) state, without giving any original authority,
that in Malabar the first three days are spent in a special room of the house;
on the fourth day the woman bathes and is then half-clean (i.e. can leave her room
but not enter the temple), until the end of the seventh day. The three days of
isolation, followed by ablutions on the fourth day, are obviously similar to the
shortest procedure laid down for Parsees in the Zend-Avesta.

Dr. Khanolkar has very kindly sent me a translation from the Sanskrit of
passages from the book Dharmasindhu, written about 1790 A.D. by Kashinath
or Baba Padhye, which is the accepted basis of Hindu ritual and religious prac-
tices in the Bombay area. The menstrual woman must have no contact with
other persons for three days and nights and is subject to numerous other prohibi-
tions during this time. On the fourth day, after cleansing the body and after
cleaning the mouth and teeth thoroughly, a full bath should be taken after sunrise
at about the time when the cows are released to go to the pastures. This bath
confers purification only for the purpose of touching ordinary household objects
without polluting them, and for attending upon the husband. It is only on the
fifth day that the woman is fit to participate in the worship of the gods and the
ancestors. Dr. Khanolkar says that these regulations"  . . . are still
observed in the Hindu society, except by modern women in big towns, who work
in schools, colleges, offices, etc., and cannot afford to remain in isolation every
month."

The lenient Hindu view of intermenstrual bleeding is of interest in comparison
with the Jewish law (p. 198). "If as a result of disease menses continue to appear
continuously, the women is not impure and remains as if she is not menstruating.
But she is debarred from participating in any acts of worship of the gods or the
ancestors, and despite continuous or infrequent discharges she shall carefully
work out a monthly period and remain under the enjoined discipline for three
nights and three days once every month."

2. Economic Conditions.

The importance of these factors is indicated by the data collected under
"Social Incidence" above, and especially by the large-scale statistics of the
Registrar-General for England and Wales. The reference by Vineberg (1919)
to the conditions under which the majority of his hospital patients in New York
lived is valuable in this connection. "When one stops to consider that of the
total number of the Jewish women 1995 had badly lacerated cervices . . .
and that they were living in the worst possible hygienic surroundings, amidst
the greatest squalor and privation, such as obtain in the lower East Side of the
Metropolis, it is truly remarkable that so few cases of cancer of the cervix were
detected amongst them."

3. Child-bearing.

The association of cancer of the cervix with parity (Lane-Claypon, 1927)
makes the low incidence upon Jewesses all the more remarkable. (For references

202

INCIDENCE OF CANCER OF UTERUS

to literature on the incidence in nulliparous women see Donaldson (1946) and
Smith (1931)). Sorsby (1931) says, "Even a superficial acquaintance with the
Jewish masses dispels the idea that Jewish women contain a large proportion of
unmarried. The reverse is true; the number of unmarried is strikingly low-
decidedly lower than among their neighbours. Nor do married women abstain
from child-bearing. Procreation is almost a matter of religious observance with
the mass of Jewesses. . . . Uterine cancer ought to be, if anything, more
common among Jewish women than among non-Jewish."

Various Talmudic writers (Epstein, 1936, 29b-30a) laid down that a father
should cause his sons to marry at 16 to 24, or 18 to 24, and that a daughter should
"be dowered, clothed and adorned, that men should eagerly desire her "; no
age is stated at which a woman should marry. The general tendency was to
give daughters rather early in marriage; this led to the Rabbinic ruling that a
man must wait for his daughter to grow up before giving her in betrothal
(Epstein, 1936, p. 205). A modern compiler (Abramowitz, 1900) states the law
of Israel on this matter thus: "After a man has arrived at the age of 18 it is his
duty to take unto himself a wife in order that he may be fruitful and multiply,
at any rate he should not pass his 20th year without having taken a wife.
Having begotten a son and a daughter who are also generative, one has fulfilled
the commandment "to be fruitful and multiply."  . . . The command "to
be fruitful and multiply" is not obligatory upon women; nevertheless a
woman should not remain single lest she be liable to suspicion. . . . It is
one of the mandates of the Sages that a man shall give his sons and daughters
in marriage immediately they approach maturity. . . ." These Jewish prac-
tices thus laid down in the Law, and described by Sorsby as prevailing at the
present day, should encourage early marriage.

The Abb6 Dubois (Beauchamp, 1906) says that" . . . A Hindu only marries
to have children, and the more he has the richer and happier he feels. . . . No
Hindu would ever dream of complaining that his family was too large, however
poor he may be   . . . barrenness in a wife is the most terrible curse that
can possibly fall on a family."

4. Early Marriage.

Lombard and Potter (personal communication) collected data from 549 cases
of cancer of the cervix, and 550 cases of cancer of the breast, in State cancer
hospitals of Massachusetts, and out of about 80 variables studied found 16 to be
significant, and of these, marriage before the age of 20 was the most strongly
associated with cancer of the cervix (Table XXXII). Thus 45 per cent of women
with cancer of the cervix, and only 16 per cent of those with cancer of the
breast, were married before that age. "Variables which might be considered
are: Earlier childbirth, multiple children, poor obstetrical service, longer duration
of married life, syphilis, immaturity of tissues at time of marriage, and excessive
hormonal stimulation." Of these factors, longer married life could be elimi-
nated by comparison with other groups. "One may surmise that a part, if not
all, of the correlation between marriage under 20 and cancer of the cervix, which
persists after elimination of the effect of the variables pertaining to pregnancy,
economics, and syphilis may be due to either immaturity of tissue or to an
oversupply of hormones. The latter may stimulate the individual to early
marriage, and may also cause malignancy."

203

204                           E. L. KENNAWAY

TABLE XXXII.-Cancer of the Cervix and Marital Status. Massachusetts.

(Lombard and Potter.)

Without chil-  Married under
Number in    Married    dren per cent  age of 20, per

series,  per cent.  of married.  cent of married.
Cancer of cervix: 2 series  .  .  549    . 960-97 7 .    93-10-8  . 44-0-49-2

,, uterus: 3 series  .  .    848    . 933-94-0  . 11 3-16-4  . 33-0-35-4
,, breast: 5 series  .  .   1509   . 81-5-84-4  . 16-1-21-7  . 11-3-22-9

,, other female organs:

1 series .  234   .    83-3    .    20-5   .    21-0
First marriages of Massachusetts

females, 1890-1939  .  .               .           .            .    18.6
Total female population; age-group

35-74.

1940 census .  .   .    .          .    83-2    .   17.2          -
1910 census .  .   .    .          .    83-1    .   15.6          -

Modi (1922) writing of Parsees says that "the marriageable age at present is
generally after 21 for the males and after 16 for the females."  "The average age
of Hindu women at marriage is 16 years, and of Parsee women 25 years."
(Khanolkar).

5. Genetic Factors.

This factor is obviously a possible one, but its action is difficult to prove or
disprove. A similar problem arises in the case of primary cancer of the liver
in the African negro (Berman, 1941; Kennaway, 1944). Data are required from
other Semitic peoples most nearly related to the Jews.* Weir and Little (1934)
consider that the maximum incidence of uterine cancer one quinquennium later
in Jewesses (Fig. 1) compared with other women, suggests a genetic basis. Cer-
tainly the age at which some forms of cancer occur is affected by genetic diffe-
rences; examples have been given in detail in another paper [Kennaway and
Kennaway (1944); xeroderma pigmentosum; familial polyposis intestini; cancer
of the breast, gastro-intestinal tract and corpus uteri in certain families]. But a
higher ratio of corpus to cervix cancer in Jewesses would have the same effect
(Fig. 3).

6. Circumcision.

Some authors (Handley, 1936, 1947) consider that no explanation of the lower
incidence of cervical cancer upon Jewesses need be sought beyond the (assumed)
difference in the bacteriali flora of the female genital tract brought about by this
factor, a matter which it would be possible to investigate. More data from
Moslem populations who practise circumcision, and from Parsees, who do not,
are obviously very desirable. Handley (1936) has emphasized the value of
statistics from Fiji, where the natives practise circumcision, in contrast to the
Hindu immigrants, in whose native country phimosis and cancer of the penis
are very common (Kennaway, 1947); certainly if these two races could receive
equal medical attention interesting results might be obtained.t

Circumcision might act in another way, not dependent upon bacterial action.
Phimosis, cancer of the penis and cancer of the cervix appear to be common in

* See "The Racial Origins of Jewish Types," by Radcliffe N. Salaman, in "Wherein I Glory,"
(London, 1948}.

t The population of Fiji in 1945 included 115,724 Fijians and 117,256 Indians (Statesman's Year-
book, 1947). Brewster (1922) states that the majority of Indians were Hindus," . . . although
there was a fair proportion of Mohammedans."

INCIDENCE OF CANCER OF UTERUS

certain areas (China, among Hindus in India; Kennaway, 1947). If it be that
material whllich accumulates under the prepuce contains carcinogenic compounds
which can cause cancer of the penis, this material might, if conveyed in sufficient
amount, cause cancer of the cervix. Plaut and Kohn-Speyer (1947) have shown
that smegma (horse), and its unsaponifiable fraction, are carcinogenic to mice.
But such transmission does not seem very likely.

7. Douching.

Comparative data for the incidence of cancer of the cervix in countries where
douching is more or less commonly performed would be of great interest. Dr.
Denoix of the Ministere de la Sante Publique has been kind enough to inform me
that no data are available from France for comparison with those from this
country.

Smith (1931) has described a positive correlation, of which there might of
course be various explanations, between douching, and especially douching with
lysol, and carcinoma of the cervix.

CANCER OF THE CORPUS UTERI.

Cancer may affect either the cervix or corpus uteri, and in mass statistics,
such as those based on death certificates, these two forms cannot be distinguished.
This is very unfortunate because it is probable that they differ in etiology. We
have no data which give directly the respective social incidence of cancer of the
cervix, and of the corpus, but information should be available from hospitals
which have separate accommodation for paying and other patients. The maxi-
mum incidence of cancer of the corpus falls about 10 years later than does that
of cancer of the cervix (Fig. 3 and Table XXXIII), and this difference may suggest,
when two batches of undifferentiated uterine cancers are compared, differences
in the proportions of the two forms. For example: (1) The data from various
parts of Bavaria given by Theilhaber (1910) shown in Tables XXIX and XXX
show that the average age at death from cancer of the uterus is earlier in the
poorer classes, and if these figures, admittedly very few in number, are put in
graphic form (Fig. 2) they show a likeness to those for the two forms of cancer
(Fig. 3), which is compatible with a higher proportion of cervix cancers in the
poorer women. (2) If one reckons the death rates of married women in England
and Wales of the four lower social classes from cancer of the uterus (Table XXXI;
Statistical Review, 1936, Text, p. 90) as percentages of the rate for Class I taken
as 100, one obtains the result shown on the right-hand side of Fig. 4. The social
difference becomes less as age advances from 35-45 to 55-65, which change is
compatible with a decreasing propoition of cervical cancers, and suggests that
it is this form which is affected by social factors.

The only authorities quoted in this paper who give any data for the incidence
of cancer of the corpus upon Jewish and non-Jewish women are Smith (1941)
and Davidsohn (1939). Smith's data (Table XIVA) show that cancers of the
corpus make up practically the same percentage of all gynaecological conditions
(7.1 and 7.8) in Jews and others, while the percentage of malignant gynaecological
tumours made up by cancers of the corpus is much higher (31.1) in Jews than in
others (10.0), owing to the smaller incidence of cancer of the cervix in Jews.

205

206

70
60
50

. 40
8

z T
z 20

20

10

E. L. KENNAWAY

.o      m,

F----

I
f..t.,.-j

j..,,~. -j

r--

i               I                I               I                I

20    30    40 '  50

Age,years

FIG. 2.-Age at death from oanoer of uterus in Bamberg, Augsburg, Wuirzburg, Erlangen,

Niirnberg and the Provinoe of Unterfranken, 1908, and in Munioh, 1906-7-8 (Theilhaber,
1910). Wohlhabende  -     ,--- Arme

ri

CL

C.

C

?

FIG. 3.-Cancer of cervix and corpus uteri (Lane-Claypon, 1927). .  -. cervix, x - - -  x

corpus (x 10)

60

70    80

I                                           I i

n f%

.v

g

L--- --

I                                I

1

S -

INCIDENCE OF CANCER OF UTERUS

Davidsohn's figures (Table XVIII) show much less difference between the per-
centages of specimens examined, made up by cancer of the corpus, and of the
cervix, in Jewesses requiring gynaecological treatment (0.83 and 0.93) than in

Q (_

Rate for social class I =100

Social
class

III

Iy O_

]g

I  o I  I

. l  l

35-         45-          55-          65-          35-   45     5-

Age,years

FIG. 4.-Social incidence of cancer of uterus at early and late ages. Mean annual death rate per

million. Married women, England and Wales, 1930-1932.

other women (3.0 and 6.4). This difference is compatible with the idea that
cancer of the cervix is the form of uterine cancer which occurs in Jewesses less
than in other women.

DISCUSSION.

1. Any comparison of the figures for Jews and non-Jews (Table XXI) with
those from the various communities in Bombay (Table XXIII) is possible only

TABLE XXXIII.-Relation of Civil State and Age to Cancer of Cervix and Corpus

Uteri in Various Countries.   (Lane-Claypon, 1927.)

Cancer.

s    ~     ~cervix.      Corpus.
Total cases from literature .  .  7986     .     389
Of these, unmarried  .  .    .     170     .      52

= 2-1%      . = 13.4%
Mean age, years.   .    .    .   45 75     .     53.3

15-- 20- 25- 30-  35-   40-   45-  50-   55-  60-  65- 70- 75- 80-
Cervix  .  .    .   3  33 269 667    1196  1474  1370  1084 691 395  134 49   13  3

Under 30.

Corpus      .   .       5        9     19   45    50   104  101  58   25   9   2     0

Totals: Cervix, 7381; Corpus 427.

cVvv

700

- 600

L._

C 500

40

,-1

4 400

-t

= 300

200

C;
-pl

Soci
clas
-v

IV

- m

I

I I

100

0

207

I

I
0

I

'. 'd                      ft pd                        0 Pd-         Ae-            ef!-

E. L. KENNAWAY

on the basis of cancers of the uterus, or of the cervix, reckoned as a percentage
of all cancers in women. Obviously these data were obtained under varying
conditions, but such as they are, they are summarized in Table XXXIV.

TABLE XXXIV.-Racial Incidence of Cancer of the Uterus.

Percentage of all cancers in women.

Cancer of uterus.

Minimum.     Maximum.

Europe and U.S.A. fNon-Jews .      .    .    .    13 9    .    25-35

(Table XXI)    ~Jews .     .    .    .    .     3.9    .     105

Cancer of cervix.

Mean.

(Hindus     .    .    .    .          44

Bombay       J Moslems   .    .    .    .           20.7
(Table XXIII)    Parsees    .    .    .    .          14 8

Indian Christians    .    .           12 7

2. The Jewish ritual has some unique features. More or less complete isola-
tion of menstruating women is practised by many other peoples and is laid downi
in the religious literature of some of these (Koran, Zend-Avesta, Dharmasindhu).
Some quite primitive peoples (Aleuts; natives of the Congo and of Australia;
for references see Ploss, Bartels and Bartels (1927)) are stated to enforce isolation
at the menstrual period for as long as 6 to 7 days. But the Jewish ritual appears
to be the only one which imposes an exact test for the cessation, and possible
recurrence, of the flow, and a post-menstrual isolation of 7 days. The 5 + 7 day
rule of the Jews is of peculiar interest in regard to the time of ovulation.

The Jewish rule suggests the following calculation. Suppose that a Jewish
woman is married at 17, and undergoes the menopause at 47, and that during 5
of these 30 years the menstrual cycle is affected by pregnancy and its sequels.
This leaves 25 years of normal cycles; if the average duration of these is 28 days,

9125

there will be  28   326 periods, and 326 X 7 = 2282 days of abstention from

intercourse after menstruation.

3. The low incidence of cancer of the cervix among Jews is the more remark-
able because they tend to be exposed to factors (early marriage; child-bearing;
in some areas low economic status) which in other communities predispose to this
form of cancer; by some means they are able in this respect to resemble the
richer women of other races.

4. The scantiness of the data available for Hindu, Moselm, Parsee and Indian
Christian women does not allow one to draw any conclusions. In the Hindu
community post-menstrual isolation of one day is combined with, in the male
population, absence of circumcision and frequency of phimosis and cancer of the
penis. The Parsees are of peculiar interest, as a one-day post-menstrual period
is combined with absence of circumcision, but the only available data are those
of the 25 cases of cervical cancer recorded by Khanolkar. These few cases
are a very uncertain basis for any conclusions, but the much higher incidence upon
Hindu women, who follow similar rules in regard to menstruation, suggest that
these particular practices do not affect the- matter, perhaps owing to the inade-

208

INCIDENCE OF CANCER OF UTERUS

quate period of post-menstrual isolation. The four-day period of abstention
possibly does not differ much from the practice of many persons whose action
in this matter is not affected by religion. We seem to have no information
about the actual procedures of isolation and purification among Moslems. The
vast area and population of China afford only a few indications that cancer of
the cervix, and of the penis, and phimosis, are all prevalent.

5. The social incidence of cancer of the cervix is obviously of great interest,
but seems not to arouse attention among gynaecologists in this country. The
social incidence upon married and single women illustrates the fundamental
importance of Stevenson's work (Registrar-General's Decennial Supplements,
1927, 1938) to students of human cancer. Perhaps the cervix-corpus junction
resembles that between the stomach and intestine in being the limit of influence
of external factors.

The term "Single Woman" is a definition of civil state which may include
more than one physiological condition. But there can be no doubt that the
social gradation shown by the 1294 cases of cancer of the uterus in single women
(Table XXXI) must depend upon factors less closely connected with sexual
functions than are those discussed above under Jewish and other rituals. Possibly
nutritional conditions are concerned here. These unique data are nearly 20 years
old. After the next census it may be possible to learn whether any change has
occurred which might be attributed to economic and dietetic differences.

6. One might make the following suggestions for further investigations:

(a) An inquiry into the incidence of cancer of the cervix in Jewish women who
observe the 12-day period, the ritual immersion being disregarded. A beginning
might be made by collecting the personal histories in individual cases.

(b) A comprehensive study by Indian workers of the unique material which
they have at hand in Hindu, Moslem, Parsee and Christian populations.

(c) Further statistical study of the social gradation of uterine cancer in married
and single women.

In all such investigations discrimination between cancers of the cervix and
of the corpus should be attempted.

SUMMARY.

1. The comparative incidence of cancer of the uterus in Jewish and non-
Jewish women has been studied in material from London, Munich, Amsterdam,
Rotterdam, Vienna, Budapest, Sweden, Palestine, New York, Chicago, Rochester,
and Philadelphia.

2. All of the twenty collections of data which have been found in the literature
show an incidence of uterine cancer which is greater on non-Jewish than on
Jewish women. These data are calculated upon several different bases and hence
are not all comparable. Seven authors express the figures for cancer of the uterus
as a percentage of all cancers in women, which percentage ranges from 28 to 14
in non-Jews, and from 10 to 4 in Jews, the mean values being 20 and 7. A similar
ratio of the order of 3: 1 is given by death rates per 100,000 in all of five instances.
Data based on admissions to hospitals show, as would be expocted, much wider
variations.

3. The low incidence of cancer of the uterus in Jews is the more remarkable
in that they are subject to some conditions (early marriage and child-bearing; in

15

209

E. L. KENNAWNVAY

some communities low economic status) which in other peoples appear to increase
the liability to this form of cancer.

4. Various degrees of isolation of the menstruating woman are, or have been,
practised over a large part of the world. The Jewish ritual appears to be the only
one which imposes an exact test for the cessation of the flow after 5 days, and for
its possible recurrence during the following 7 days. This 12-day period of absten-
tion from intercourse is of interest in regard to what is now known of the usual
time of ovulation: The Jewish ritual immersion is probably of no direct signifi-
cance in this matter.

5. The Hindu and Parsee women of the Bombay area follow rules, involving
a menstrual isolation of 3 + 1 days only. The very scanty data available (25
cases only) show a much lower incidence of cancer of the cervix on Parsees. This
might signify that so short a post-menstrual period does not affect the matter
one way or the other, anid this difference in liability to cancer must then be due
to other factors. The similar social incidence in both married and single
women in England and Wales shows that such factors exist.

6. Cancer of the uterus appears to be prevalent in some peoples (Hindus,
Chinese) among whom phimosis and cancer of the penis are also common, while
the Moslem women of India show a lower incidence. But any attribution of this
difference to the practice of circumcision by Moslems is very doubtful in view of
the similar low incidence on peoples (Parsees, Indian Christians, possibly some
Dutch) in whom this factor is absent. But the numerical data on this matter
are still quite inadequate.

7. The only numerical data on the social incidence of cancer of the uterus
appear to be those from Bavaria 40 years ago, and from England and Wales, after
the last census, in 1930-32. The liability to cancer of the uterus increases with
descent in the social scale, and, in England and Wales, this is true of both married
and single women.

8. The unavoidable mixture, in large-scale statistics, of cancers of the cervix
and of the corpus uteri, is unfortunate, as these two forms probably differ in
etiology. The difference of 8-10 years in the maximum age-incidence of the two
sometimes enables suggestions to be made of the relative proportions of the two
in numerical data.                                  -

9. The unique data of the Registrar-General upon the social incidence of
cancer of the uterus in married and single women suggests a factor less closely
connected with sexual functions than are those discussed above in connection
with various rituals.

10. The data collected in this paper suggest the existence of two factors which
may increase thle incidence of cancer of the uterus, namely:

(1) A factor which is opposed by the Jewish practice of abstention from
intercourse during most of the first half of the ovulatory cycle; and

(2) A factor which is intensified in both married and single women by
descent in the economic scale.

I wish to express my thanks to the British Empire Cancer Campaign, the
Anna Fuller Fund, and the Jane Coffin Childs Fund for grants. I am indebted
to Rabbi Dr. I. Epstein, Principal, Jews' College, London, for information about
Jewish literature, and for the loan of books. I wish to thank also Dr. V. R.
Khanolkar, for unique unpublished data from Bombay, and for translations from

210

INCIDENCE OF CANCER OF UTERUS            211

Hindu literature. I am indebted also to Dr. Heyman and Dr. Karplus for-data
from Sweden, and from Palestine, respectively; to Dr. Hannah Billig, Dr. M.
Landau, and Dr. Arnold Sorsby for information; to Mr. J. A. Heady, Statistician
to this Hospital, for much heIp in calculations; and to my secretaries, Miss
Fenning and Miss Atkin.

REFERENCES.

ABRAMvOWITZ, B.-(1900) 'The Law of Israel.' (Trans. S. D. Aaronson). New York

5660.

ADAMOWICKOWA, S.-(1929) 'Cancer in Warsaw as a Racial Problem.' Nowotwory:

4 (No. 1).-(1932) War8z. Carsop. lek., 9, 1145 (Dec. 8), and 1170 (Dec. 15),
quoted by Davidsohn (1939).

AUERBACH, E.-(1908) Z. Demogr. Statist. Jud., quoted by Davidsohn (1939).
BERMAN, C.-(1941) S. Afr. J. med. Sci., 6, 145.
BRAITHWAITE, J.-(1901) Lancet, ii, 1578.

BREWSTER, A. B.-(1922) 'The Hill Tribes of Fiji.' London.
DAVIDSOHN, I.-(1939) Med. Leaves, 2, 19.

DONALDSON, M.-(1946) Brit. med. J., i, 291.

DUBOIS, J. A.-(1906) "Hindu Manners, Customs and Ceremonies." (Transand ed.

HI. K. Beauchamp.) Oxford.

EPSTEIN, I.-(1936) 'Kiddushin, The Babylonian Talmud. Seder Nashim.' (Ed.

I. Epstein, Trans. H. Freedman.). Vol. I. London.-(1948) "Niddah. The
Babylonian Talmud. Seder Tohoroth Niddah." (Ed. I. Epstein, Trans. I. W.
Slotki.) London.

FISHBERG, M.-(1902) 'Jewish Encyclopedia,' New York and London, 3, 529.
HANDLEY, W. S.-(1936) Lancet, i, 987.-(1947) Brit. med. J., ii, 841.
HOFFMAN, F. L.-(1933) Amer. J. Cancer, 17, 142.
HORWITZ, A.-(1927) Surg. Gynec. Obstet., 44, 355.

HURWITZ, H.-(1921) "The Well of Purification."  Leeds.
JHAVERI, K. M.-(1910) J. anthrop. Soc. Bombay, 9, 217.

'Jewish Encyclopaedia.'-(1891) (New York and London). Ablution; Andernach;

Bath.

KAUFFMANN, F.-(1926) Zbl. Gynmik., 50, 198.

KENNAWAY, E. L.-(1944) Cancer Res., 4, 571.-(1947) Brit. J. Cancer, 1, 335.
IdeMn AND KENNAWAY, N. M.-(1944) Yale J. Biol. Med., 17, 139.

LANE-CLAYPON,. J. E.-(1927) Reports on Public Health and Medical Subjects No. 40.

London (H.M. Stationery Office).

MAXWELL, J. L.- (1929) 'The Diseases of China,' 2nd edition, p. 479.

MILLER, D.-(1930) 'The Secret of the Jew. His Life-His Family.' 6th ed. Oak-

land, California.

MODI, J. J.-(1922) 'The Religious Ceremonies and Customs of the Parsees.' Bombay.
NATH, V., AND GREWAL, K. S.-(1935) Indian J. med. Res., 23, 149.
PELLER, S.-(1931) Z. Krebsforsch., 34, 128.

PLAUT, A., AND KOHN-SPEYER, A. C.-(1947) Science, 105, 391.

PLOSS, H., BARTELS, M., AND BARTELS, P.-(1927) 'Das Weib in der Natur und

Volkerkunde.' 11th edition. Berlin. Vol. I, pp. 694-778.
PREUSS, J.-(1923) 'Biblisch-talmudische Medizin.' Berlin.

Registrar-General's Statistical Review of England and Wales (1935-1939), Tables,

Text. H.M. Stationery Office, London.

Registrar-General's Decennial Supplement. England and Wales, 1921. Part II (1927)

H.M. Stationery Office, London.

Registrar-General's Decennial Supplement. England and Wales, 1931. Part IIa (1938).

H.M. Stationery Office, London.

212                     W. L. HARNETT

SALE, G.-(1861) 'The Koran, commonly called the Alcoran of Mohammed.' London.
SANDERS, J.-(1916) Ned. Tijdschr. voor Geneesk., Tweede Helft A. 8, 604.

SMITH, F. R.-(1931) Amer. J. Obstet. Gynec., 21, 18.-(1941) Ibid., 41, 424.
SORSBY, M.-(1931) 'Cancer and Race.' London.
Statesman's Yearbook.-(1947) ' Fiji.' London.

SUTHERLAND, I.-(1947) Brit. J. soc. Med., 1, 126.

THEILHABER, A.-(1909) Miinch. rmed. Wschr., 58, 1271.

Idem AND GREISCHER, S.-(1910) Z. Krebsforsch., 9, 530.
THEILHABER, F.-(1910) Ibid., 8, 466.

VINEBERG, H. N.-(1919) 'Contributions to Medical and Biological Research, dedicated

to Dr. William Osler in honour of his 70th birthlday,' 2, 1217. New York.
WEIFR, P., AND LITTLE, C. C.-(1934) J. Hered., 25, 277.

ZEND-AVESTA.---Part I. 'The Vendidad.' Sacred Books of the East. Ed. F. Max

Muiiller, Trans. J. Darmesteter (1880). Oxford.

				


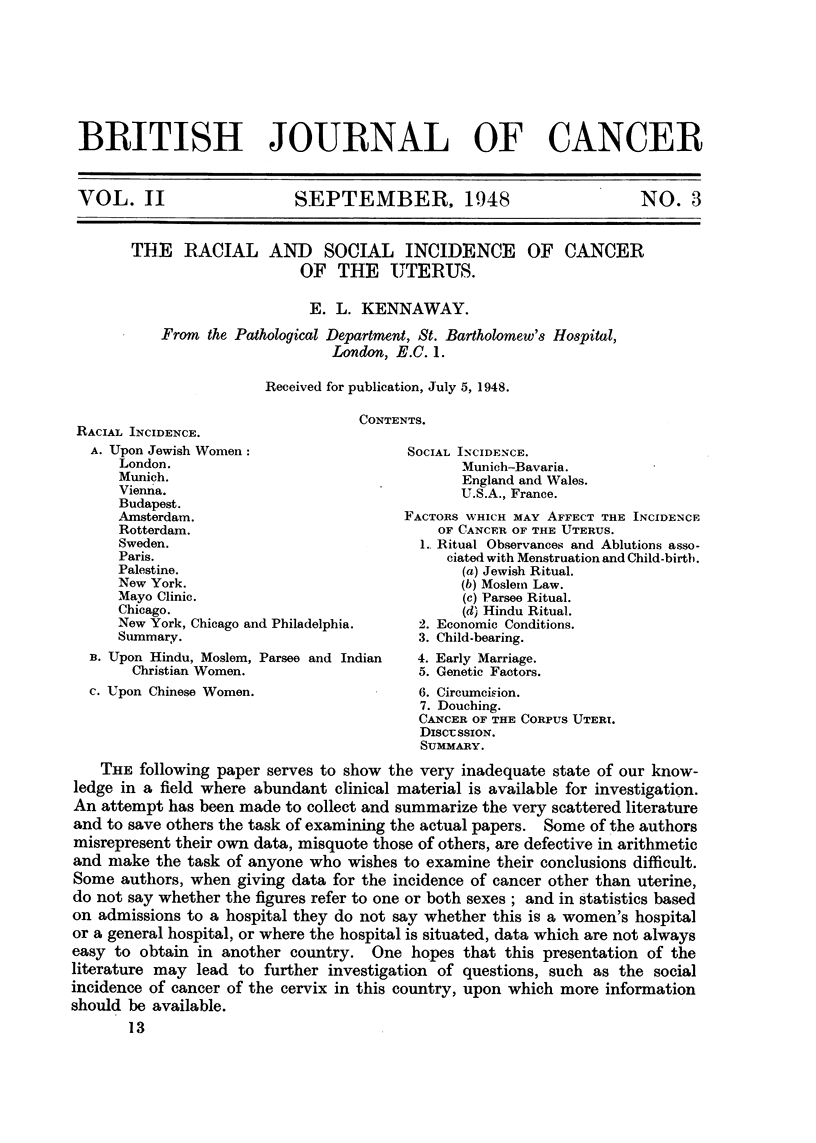

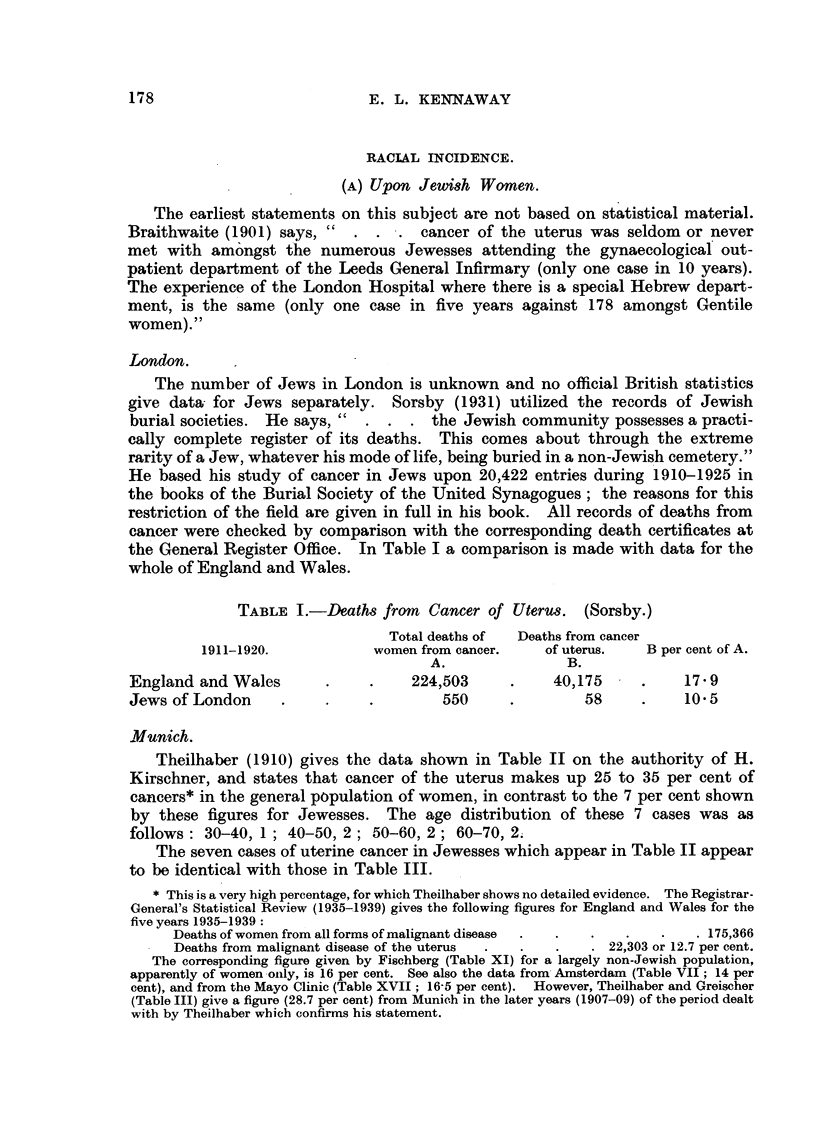

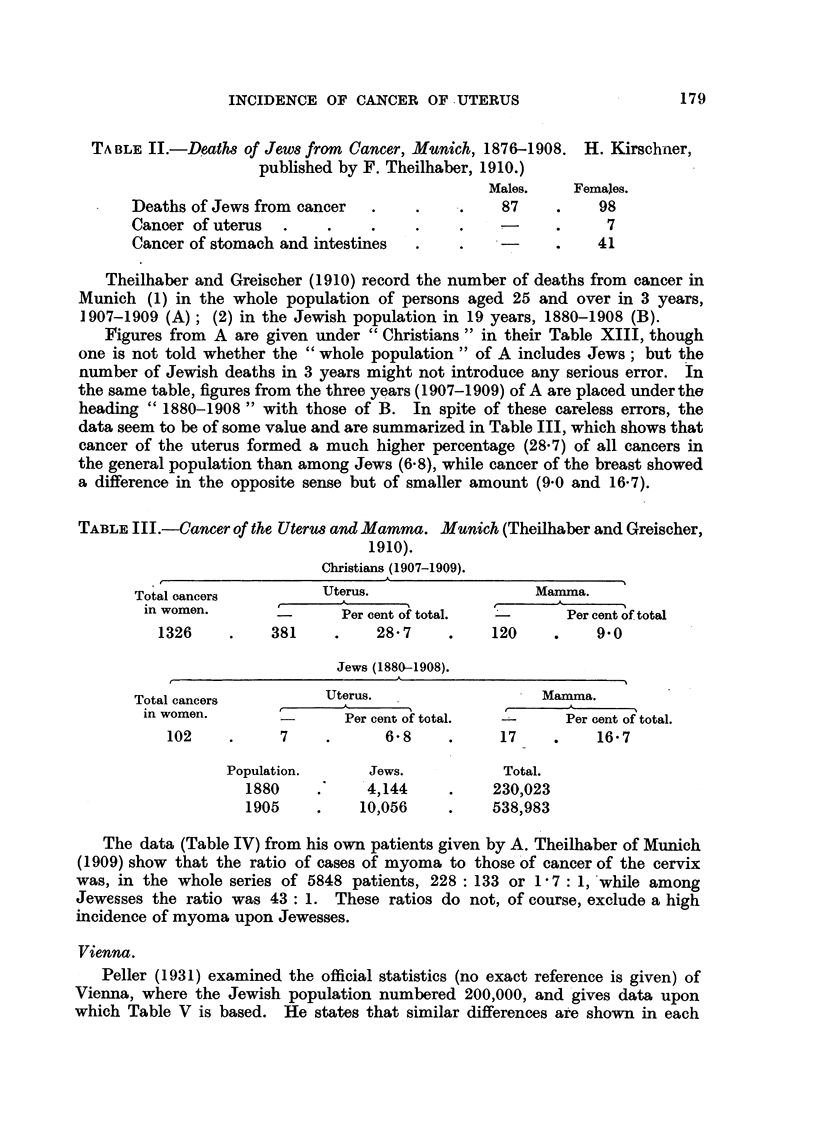

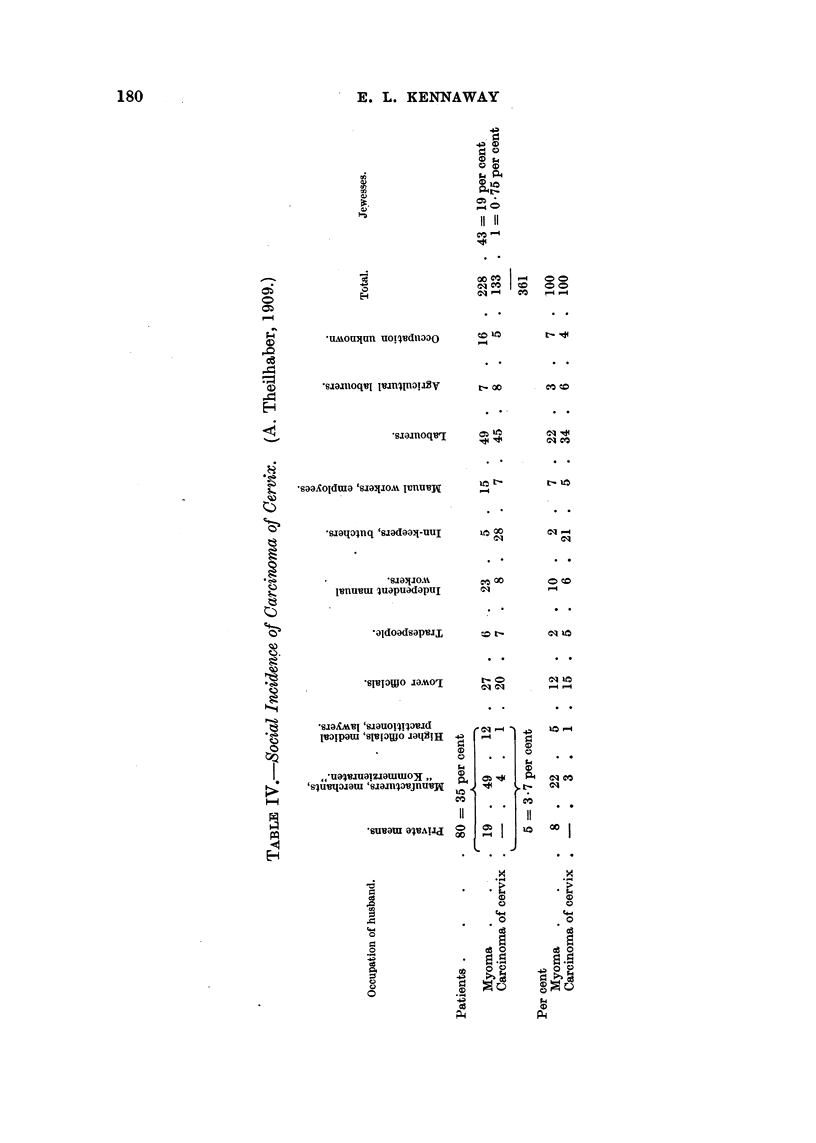

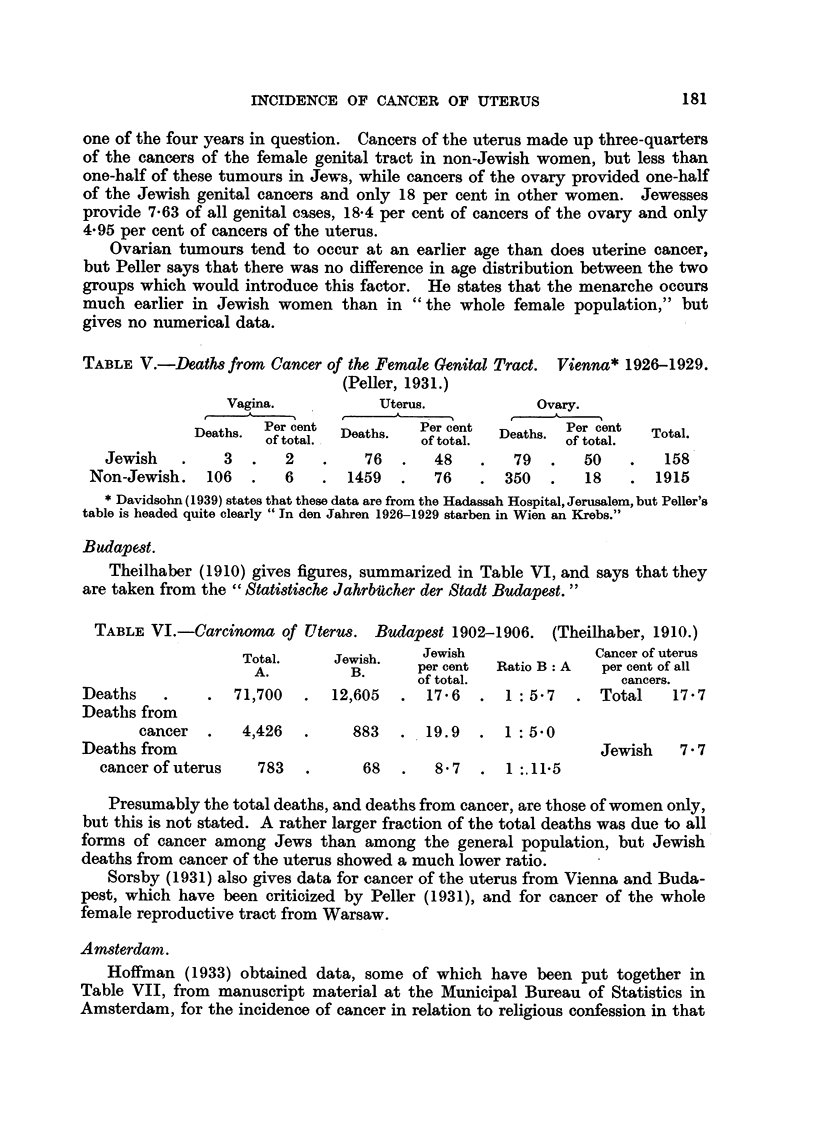

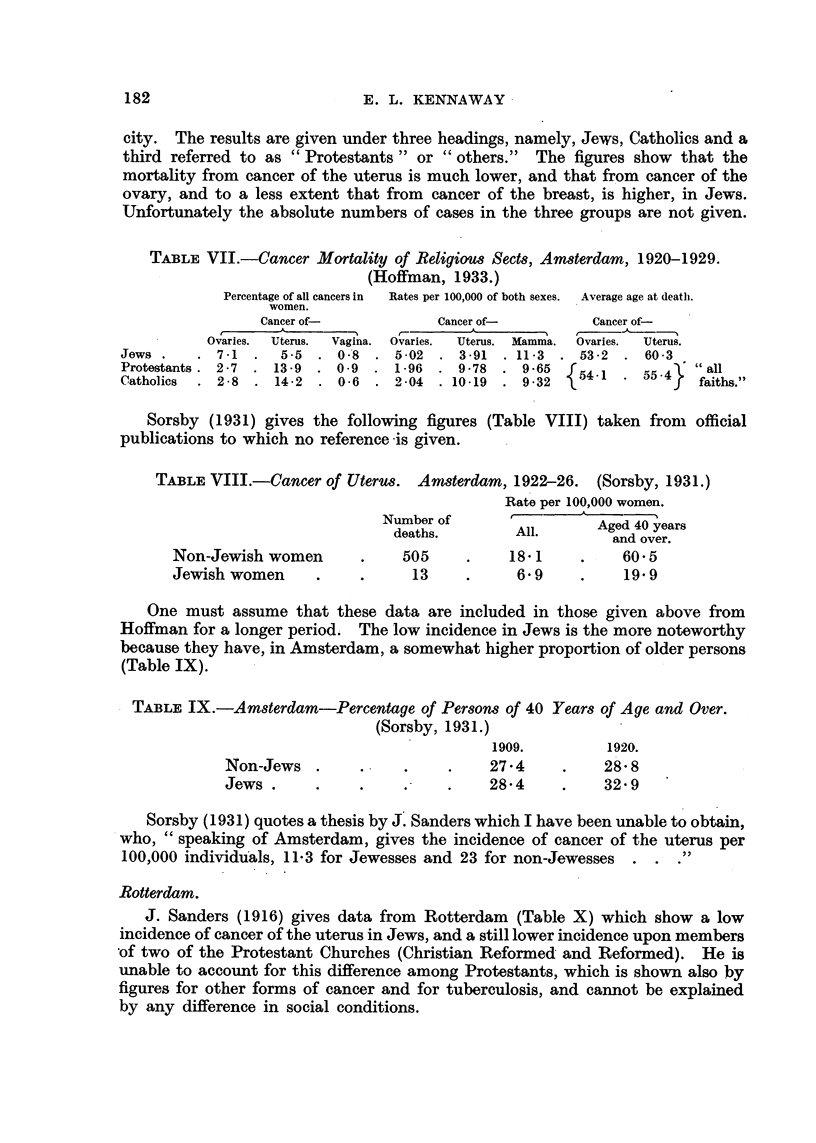

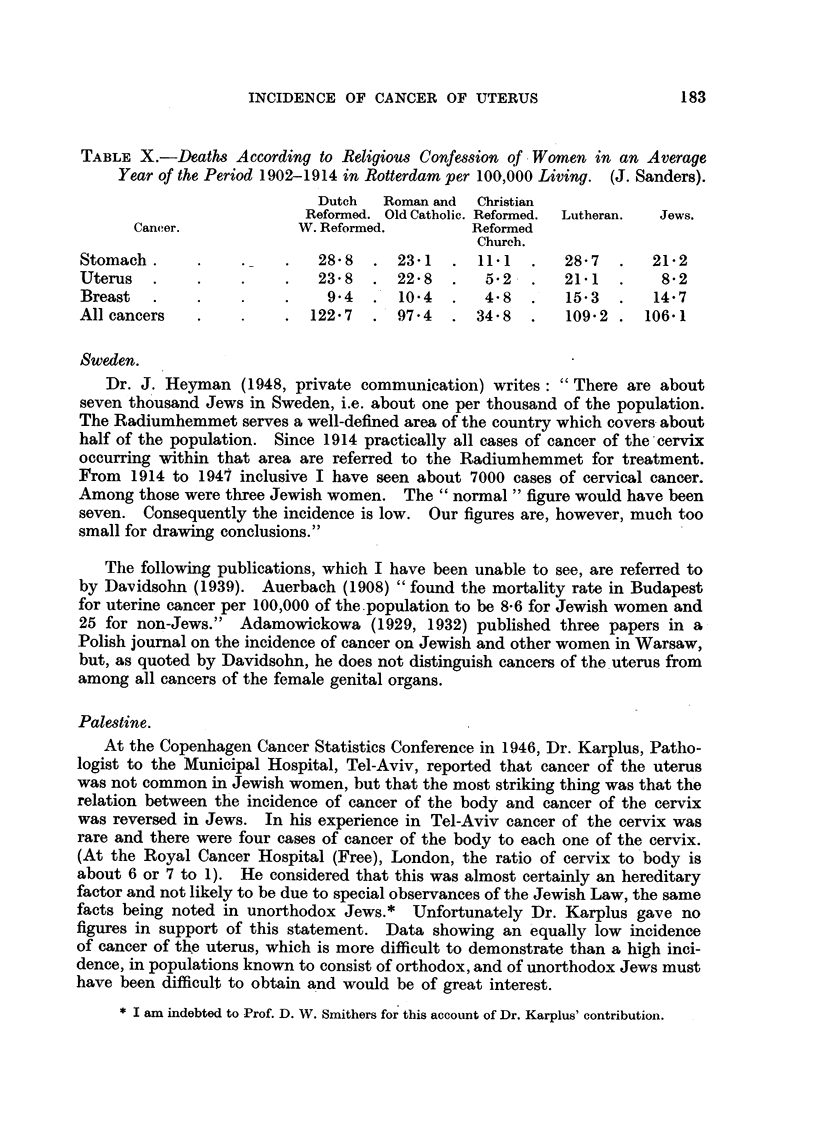

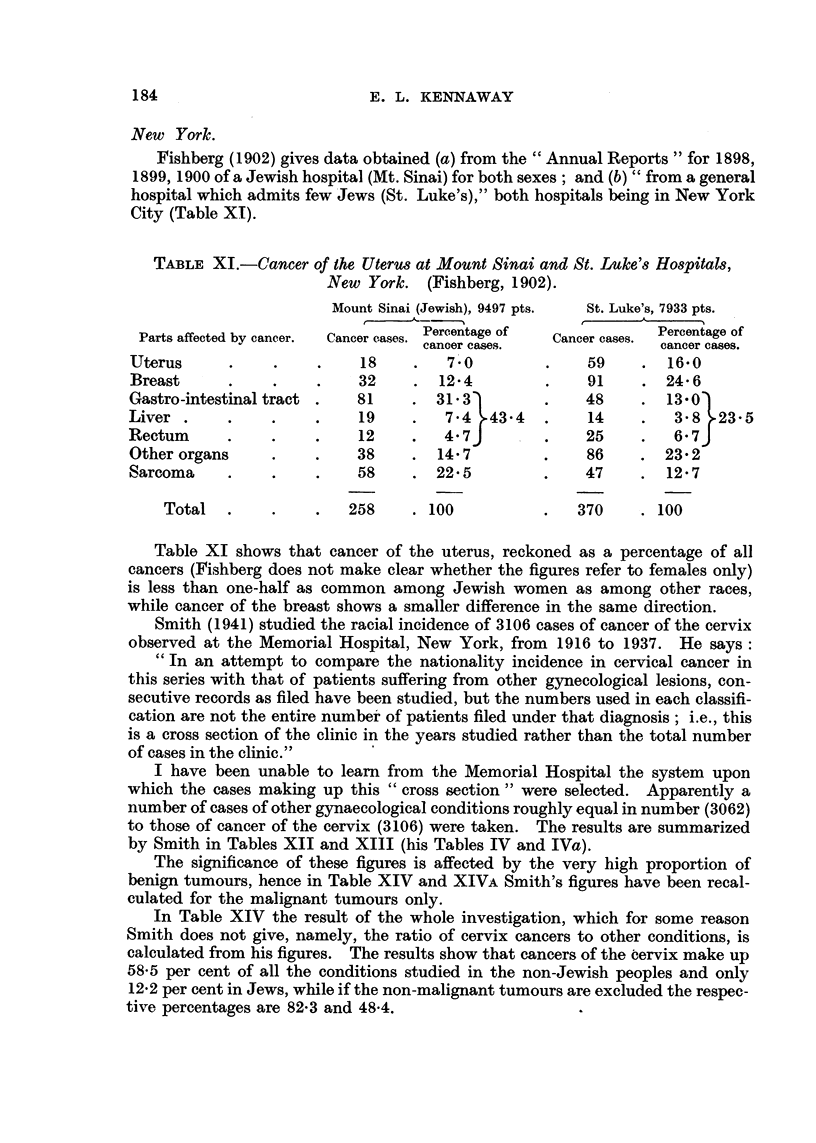

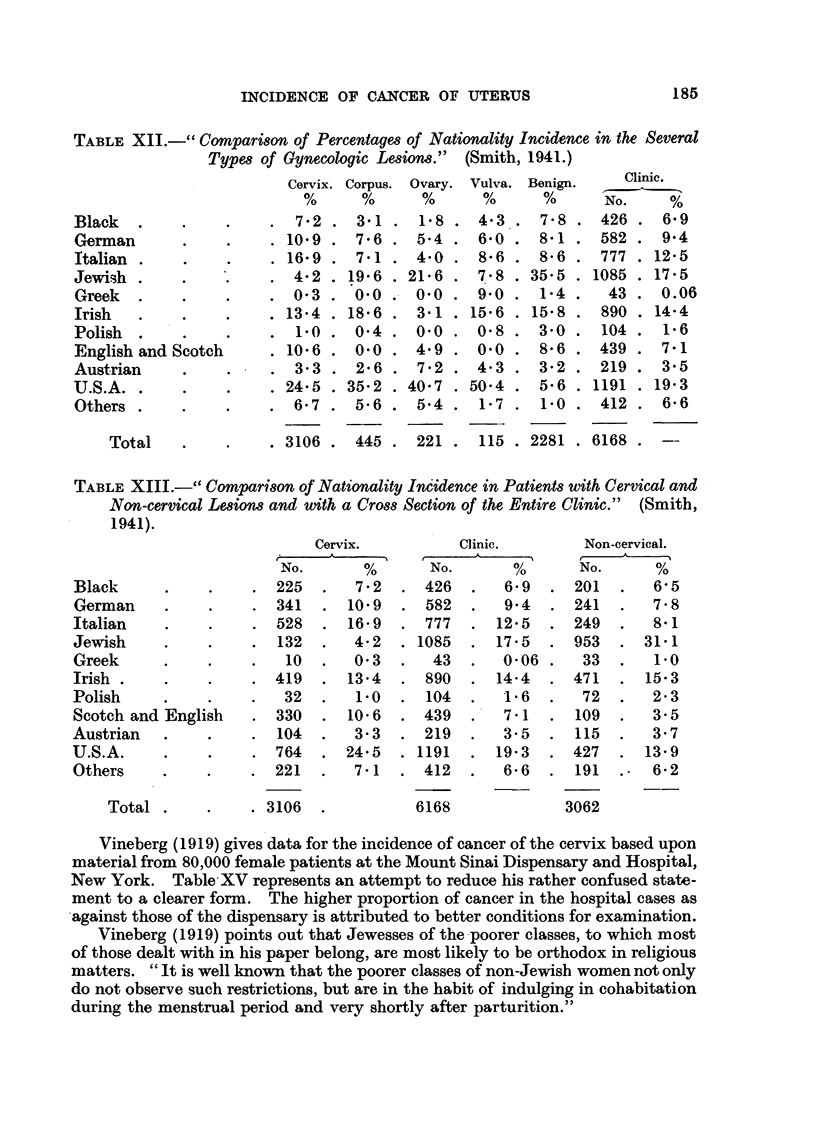

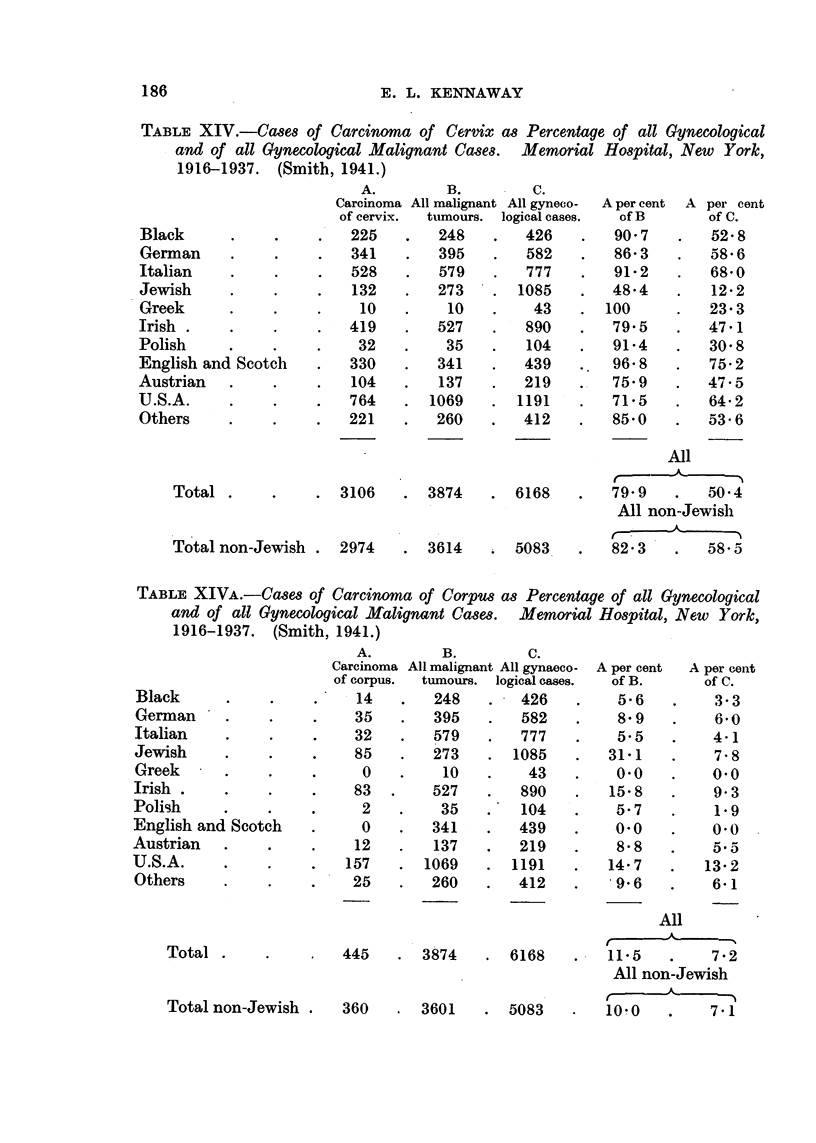

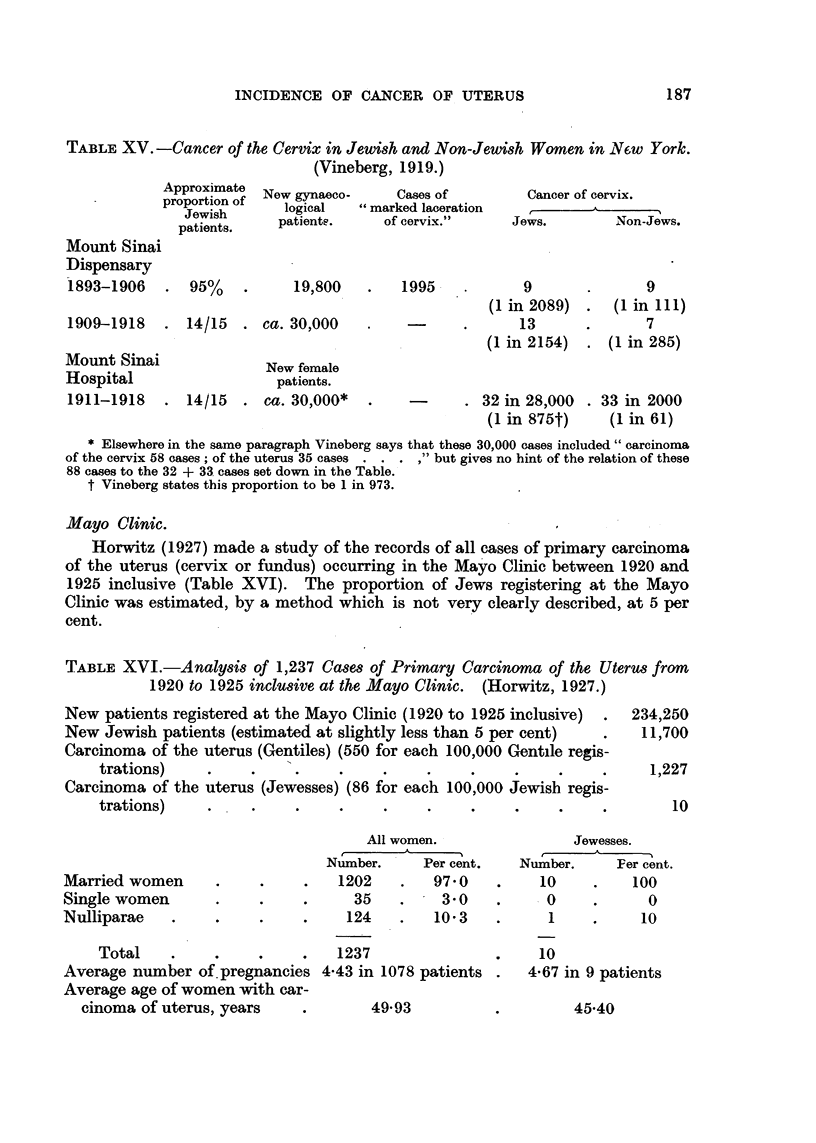

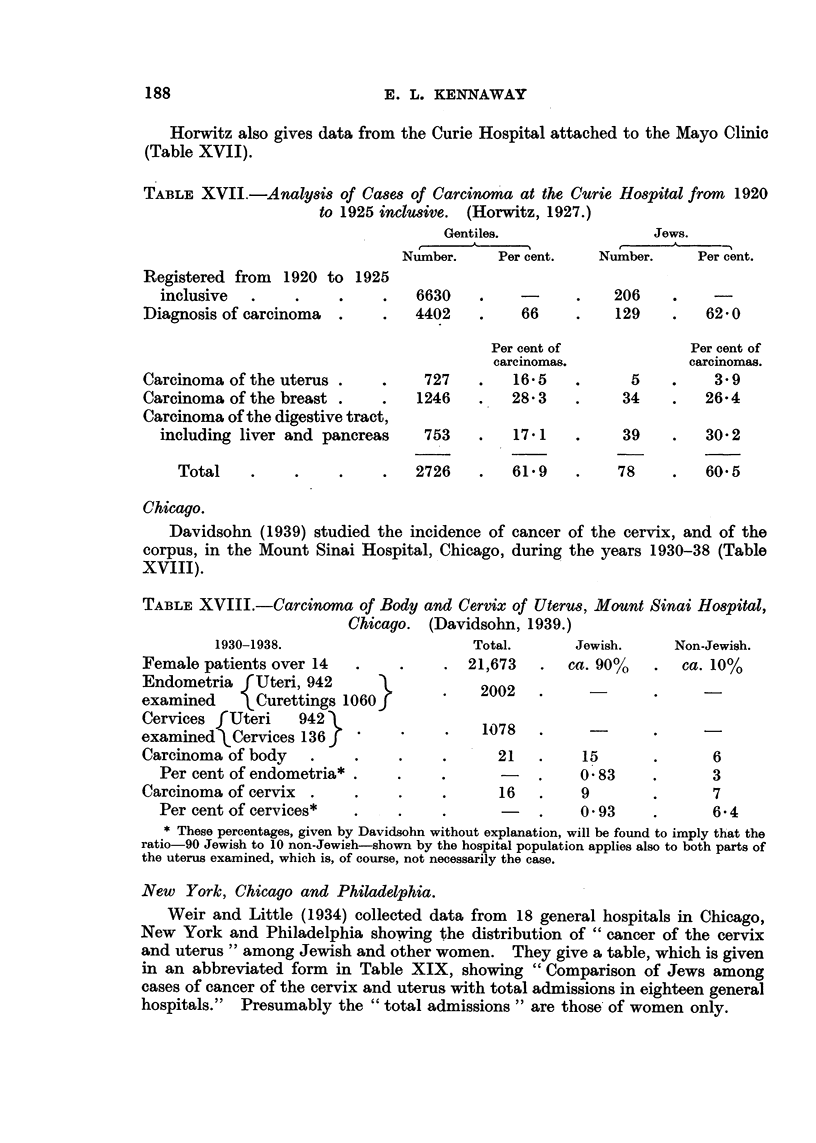

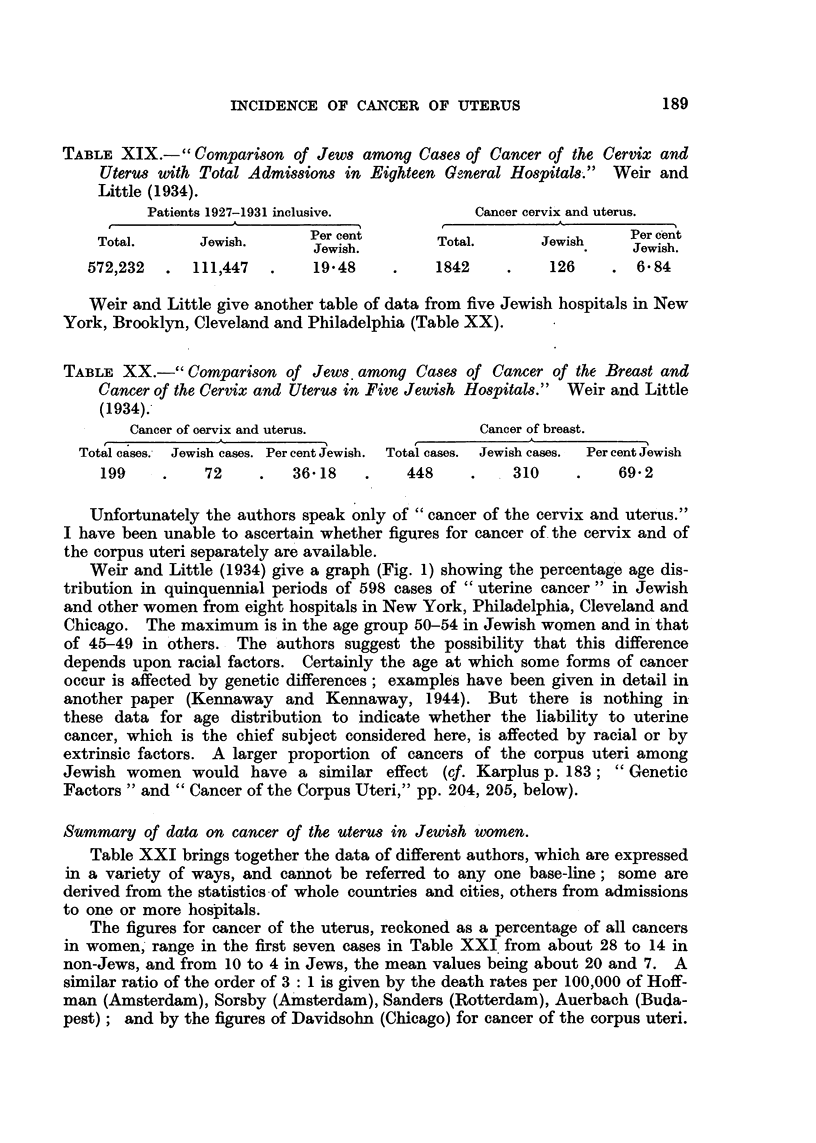

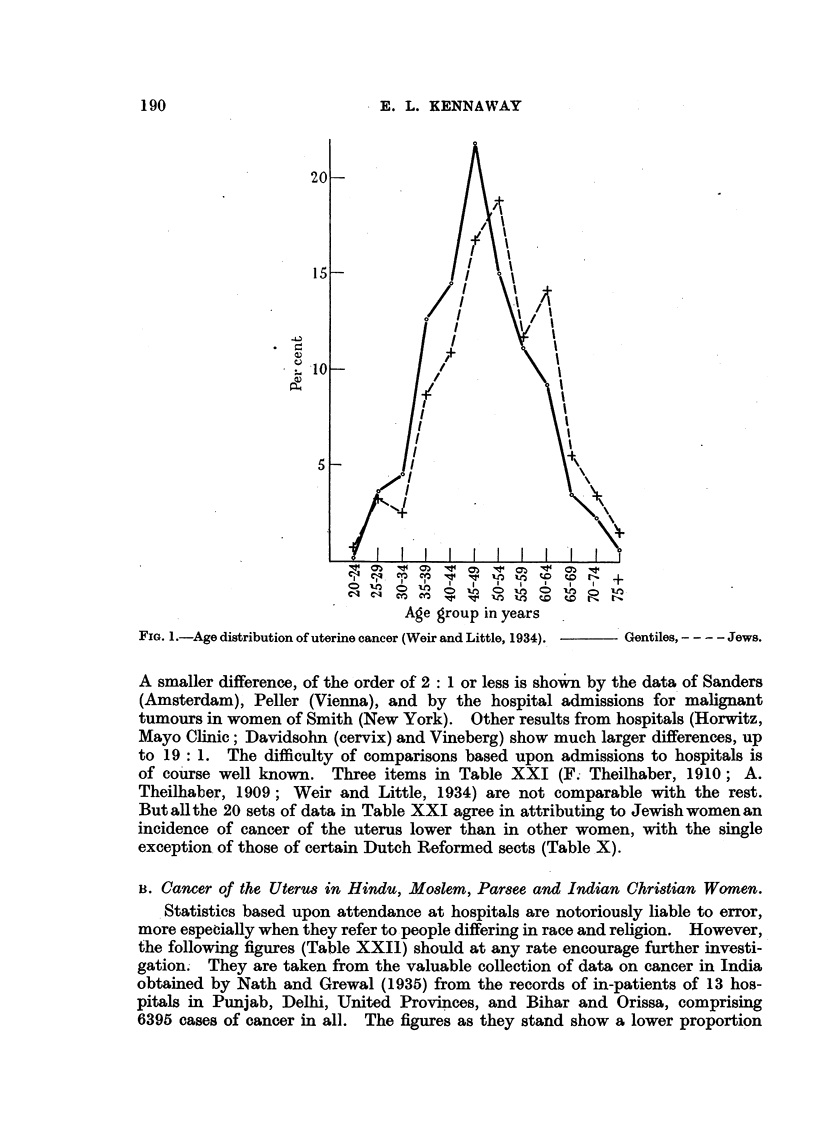

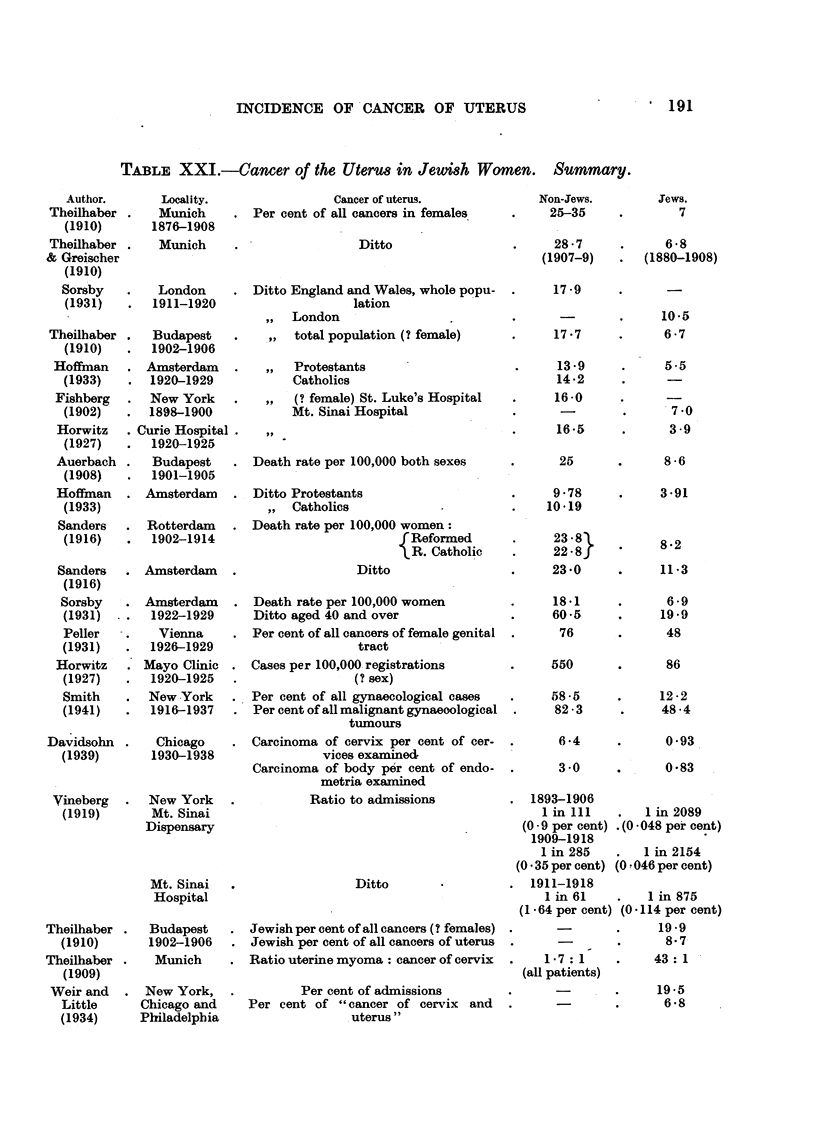

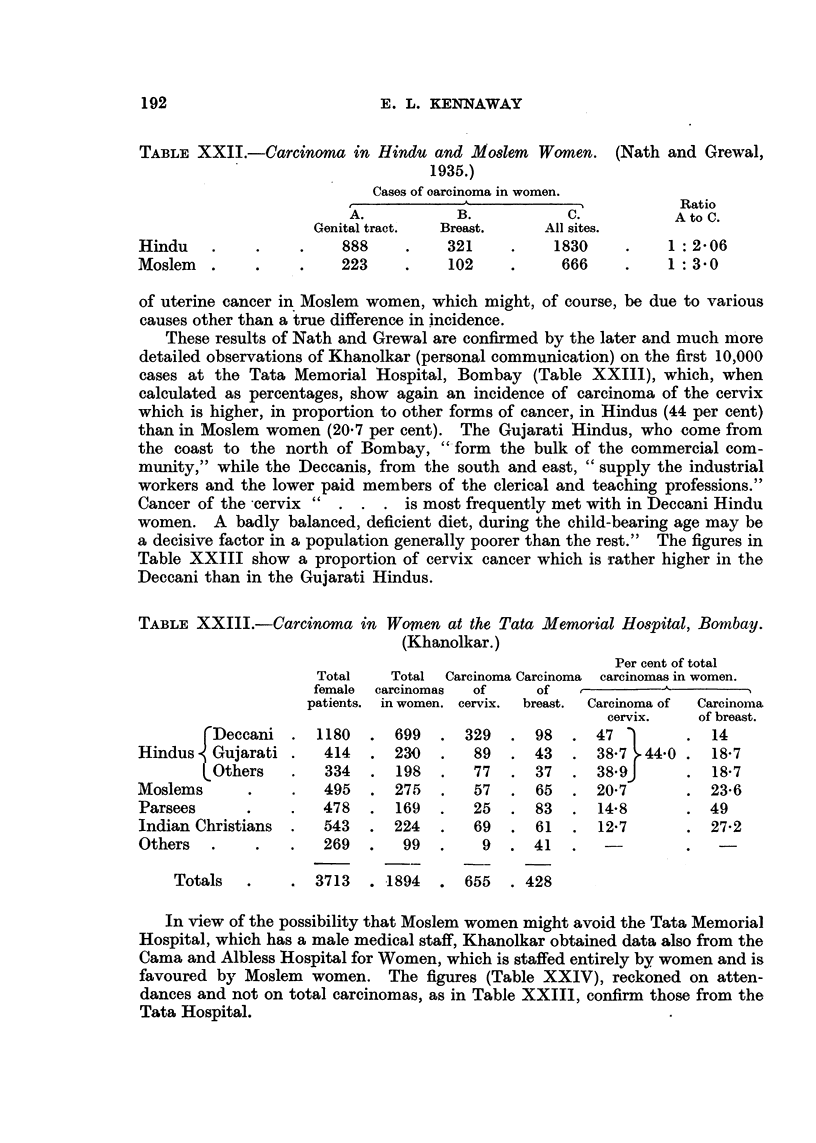

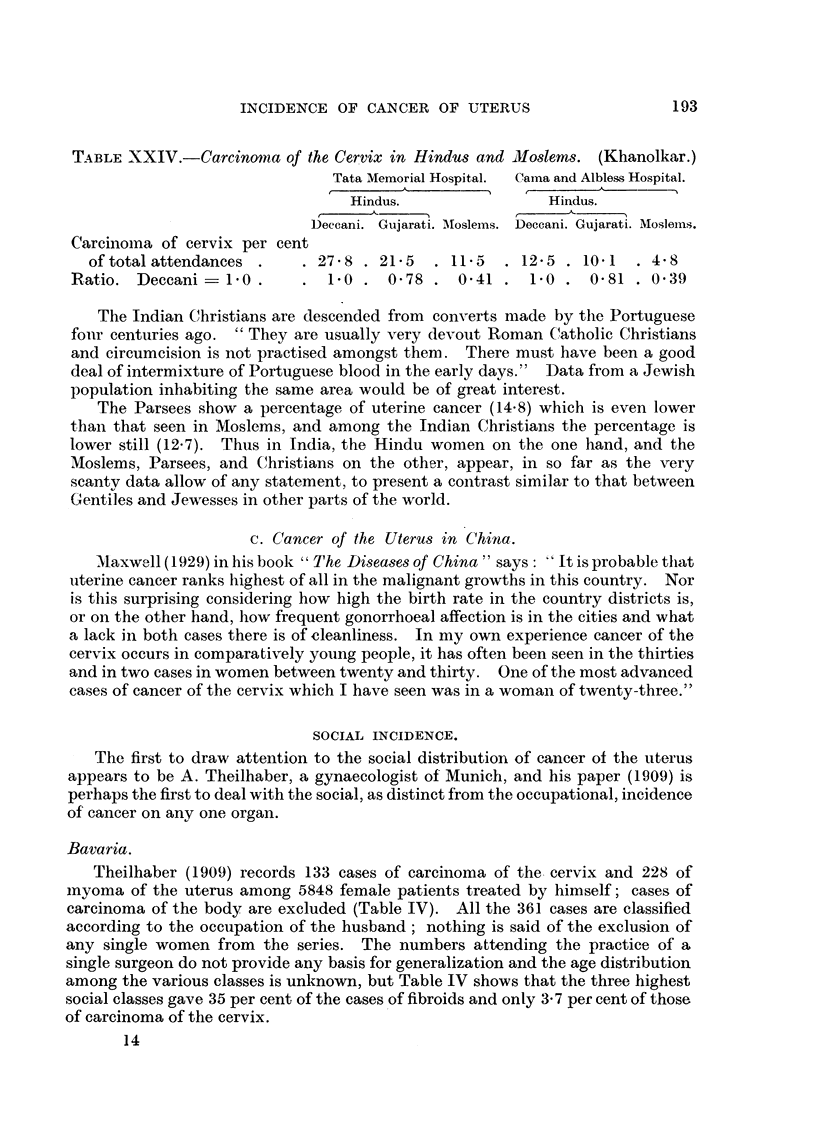

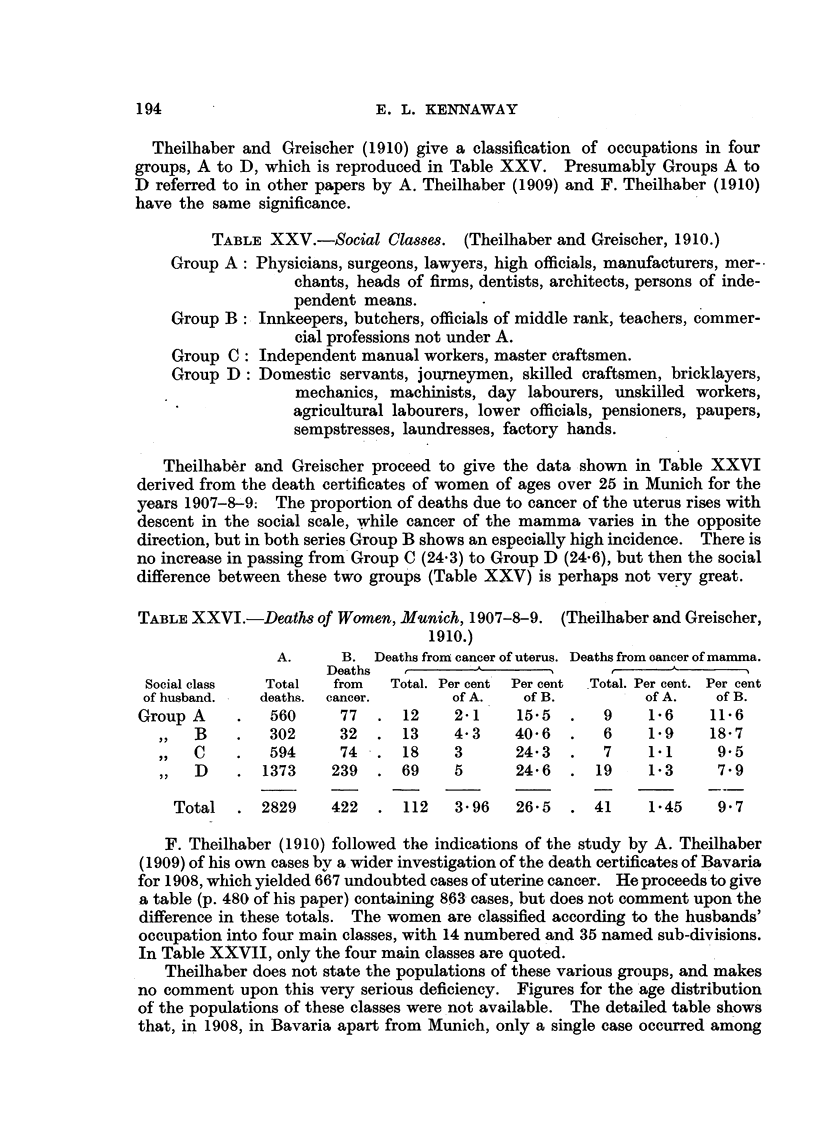

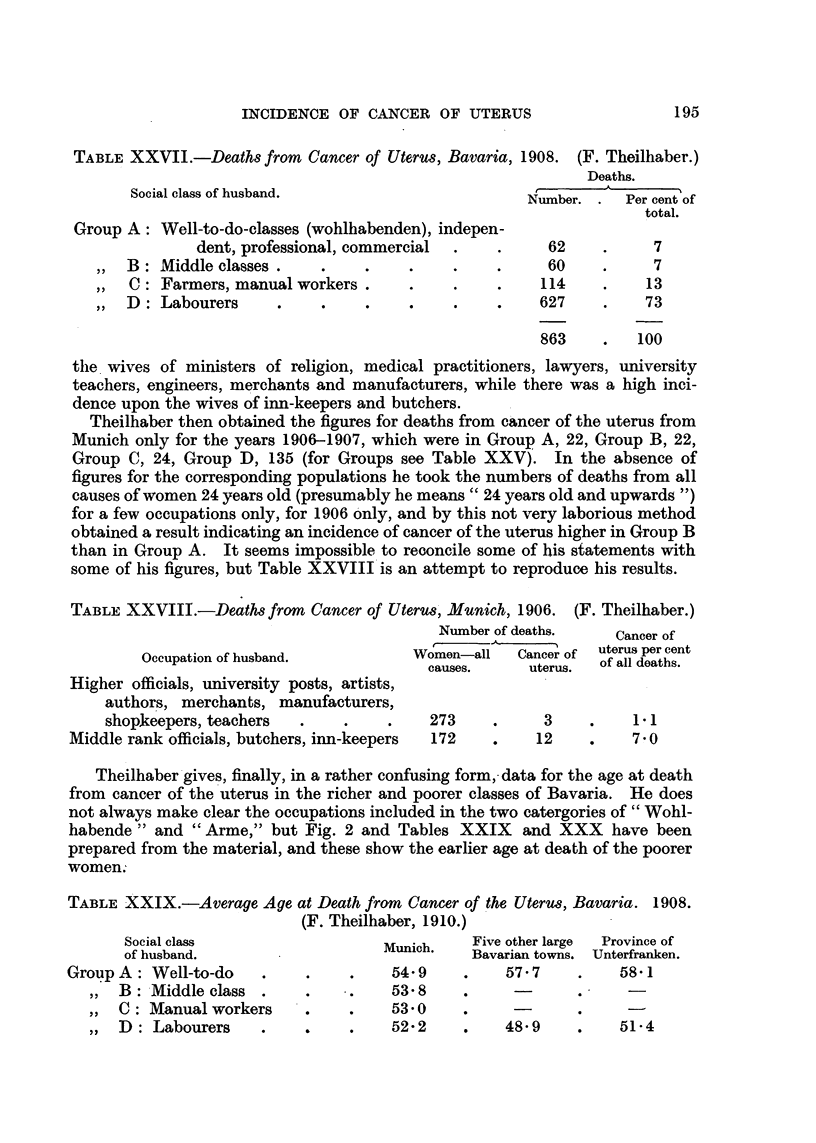

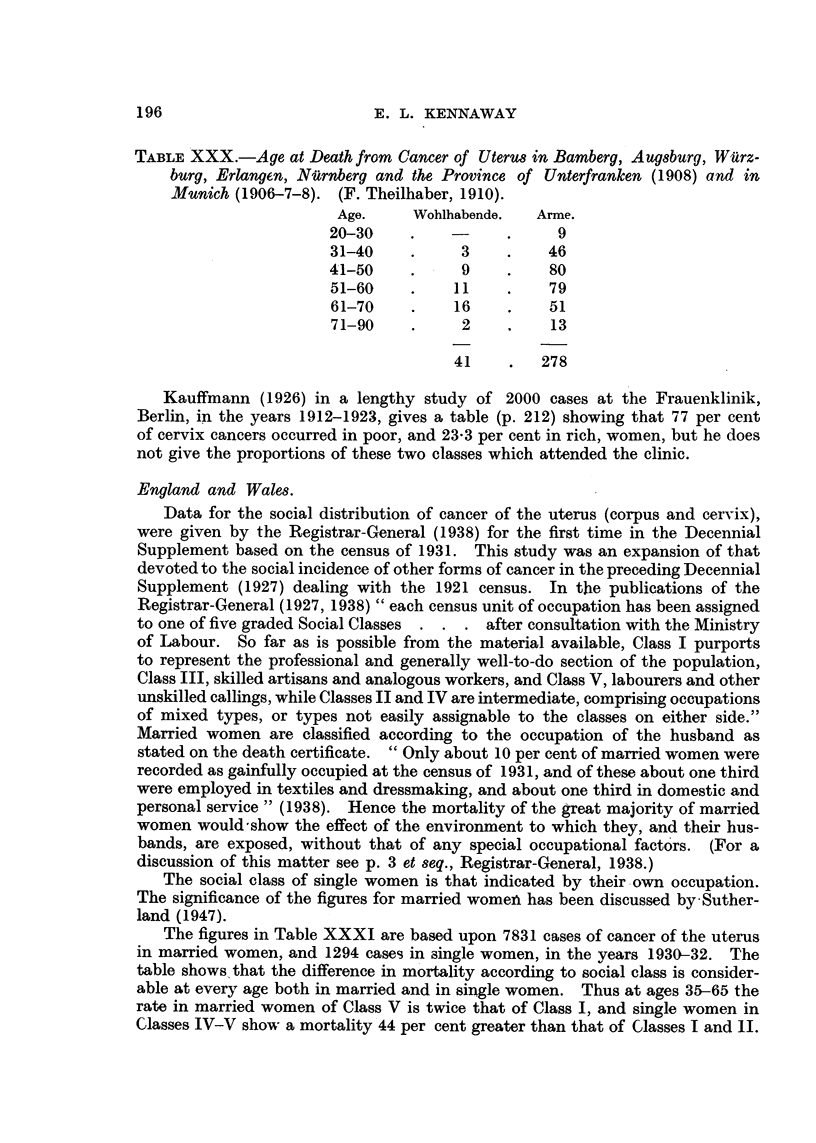

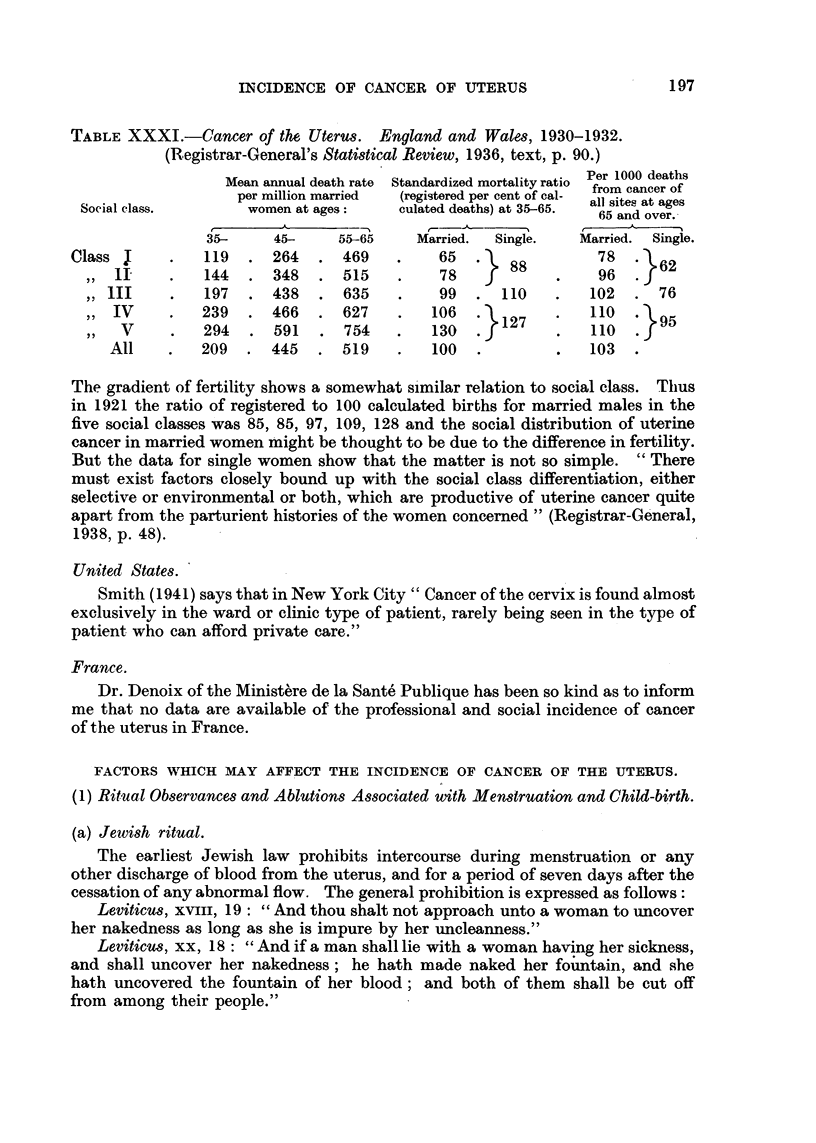

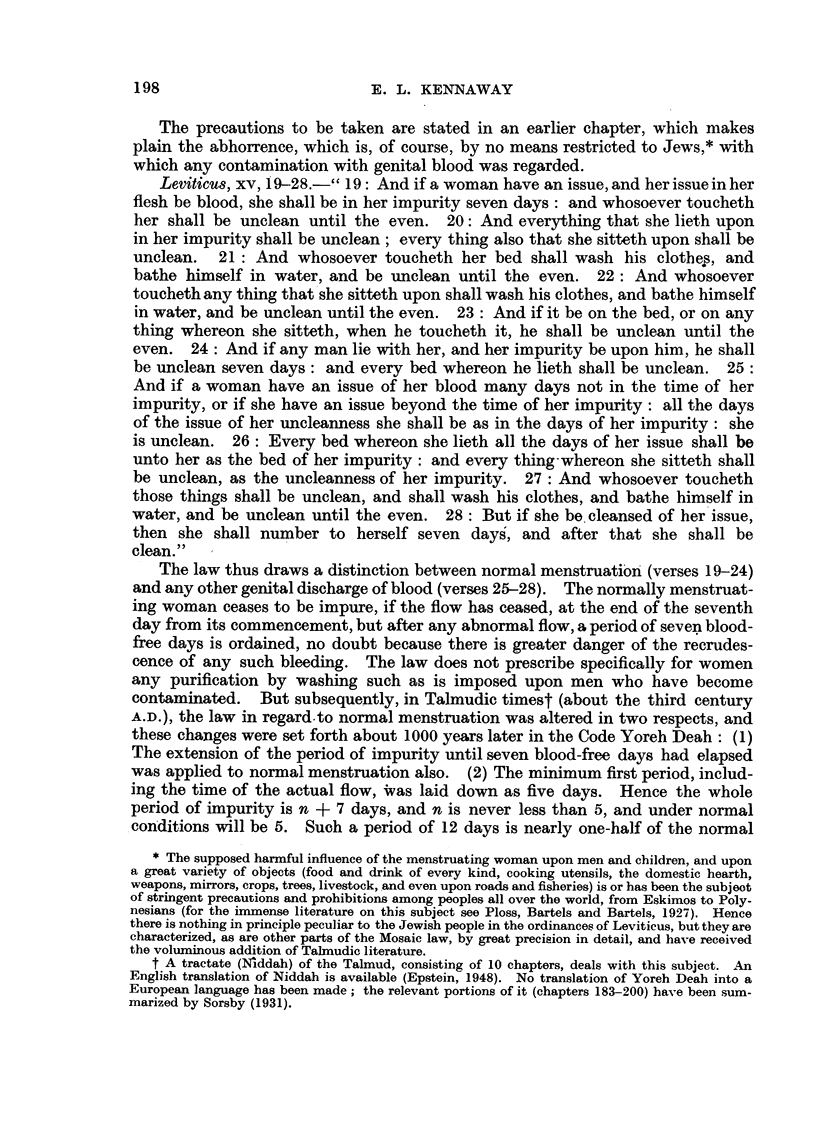

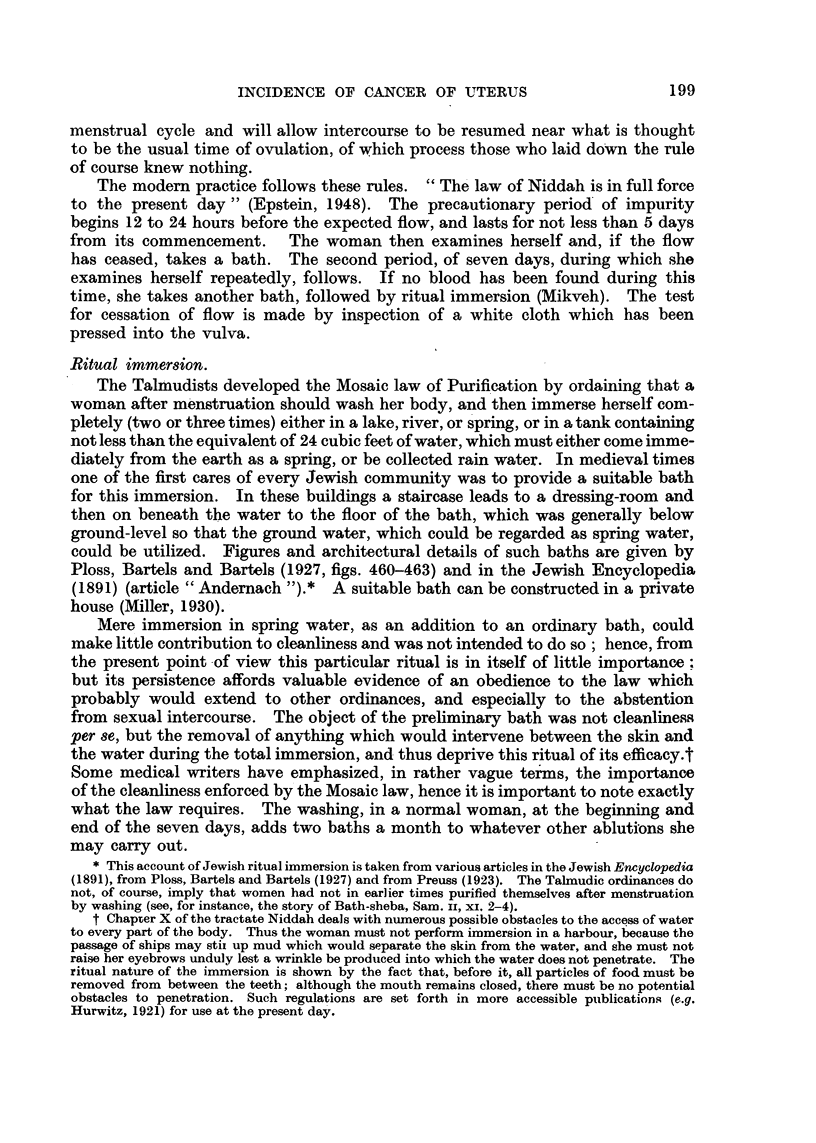

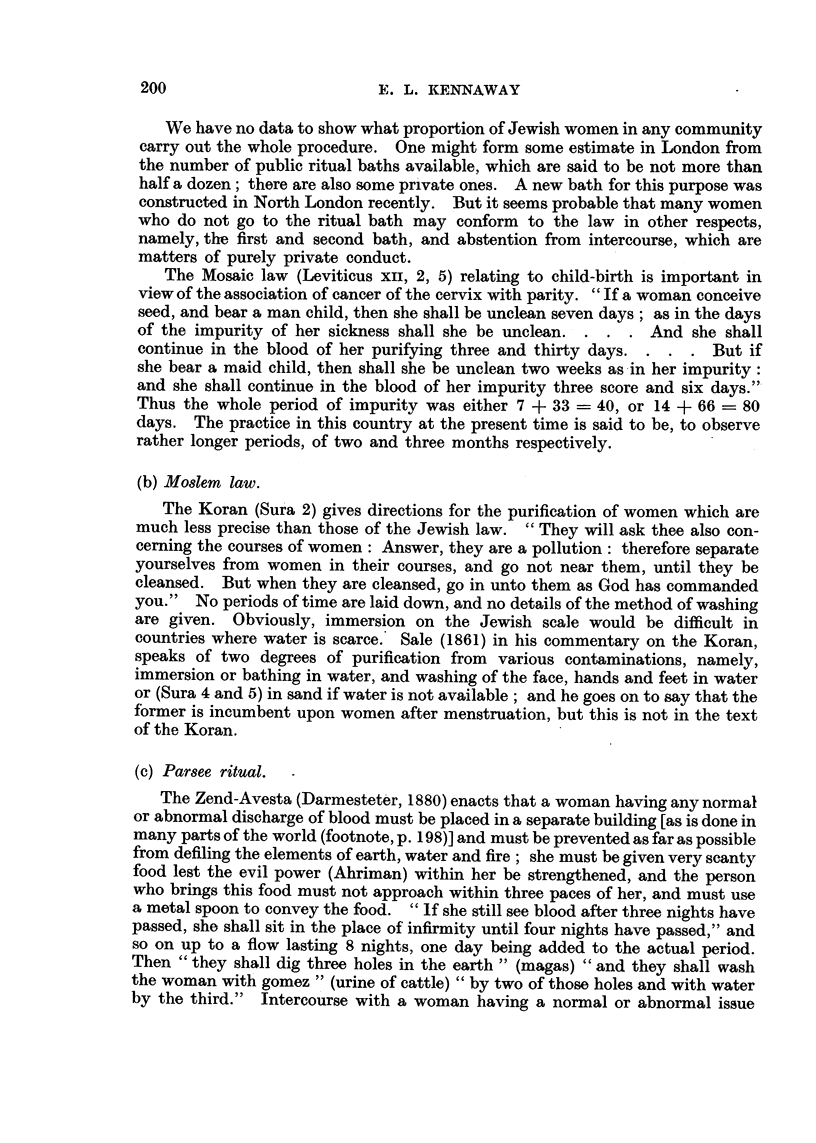

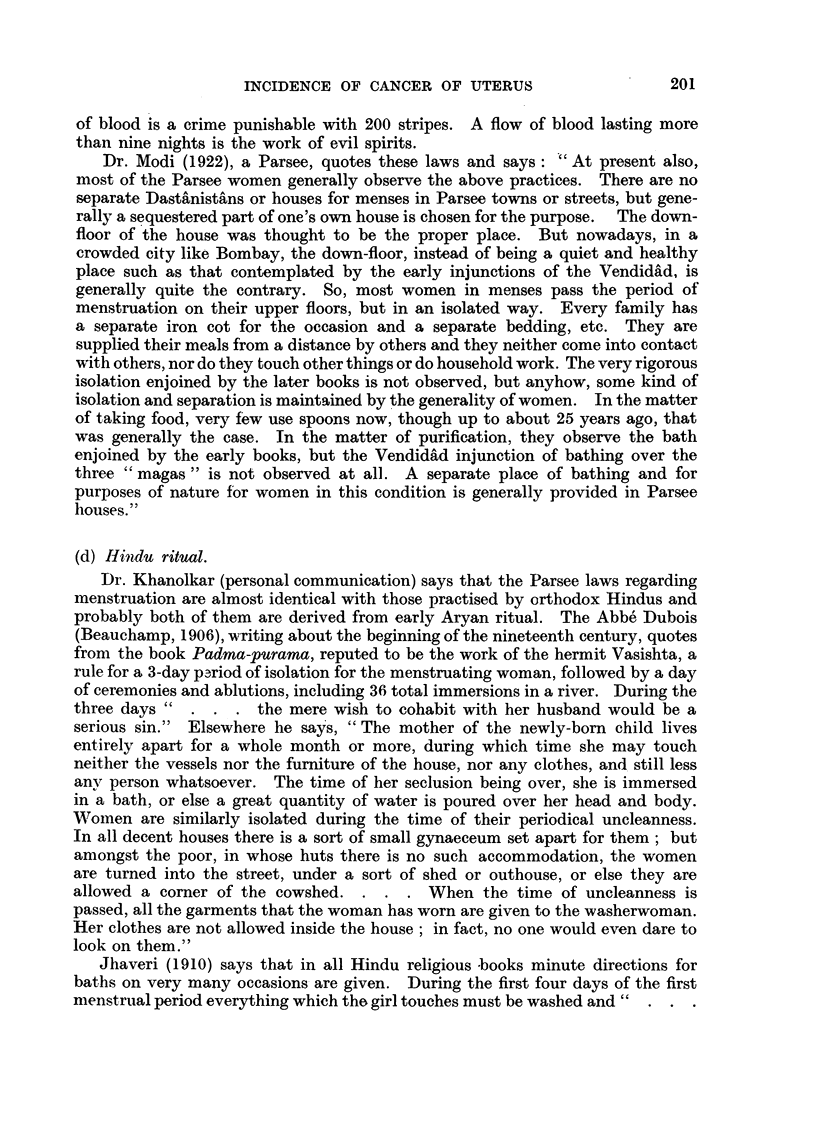

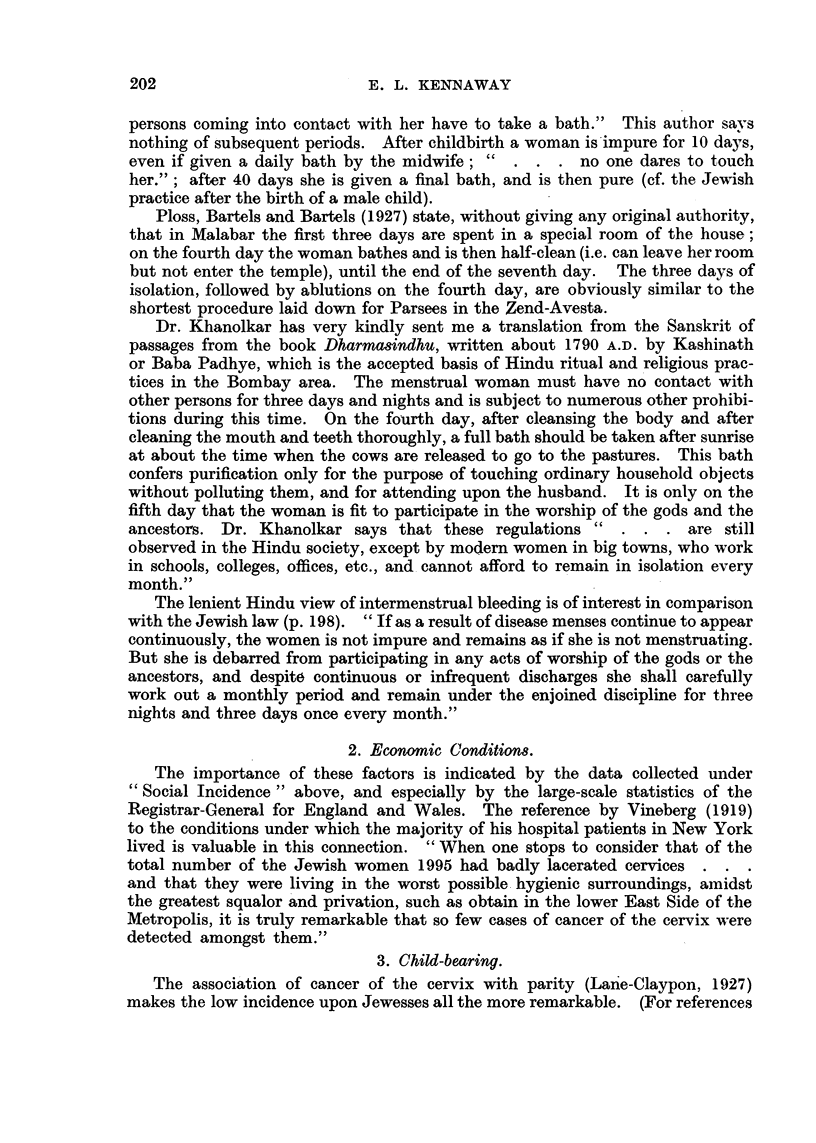

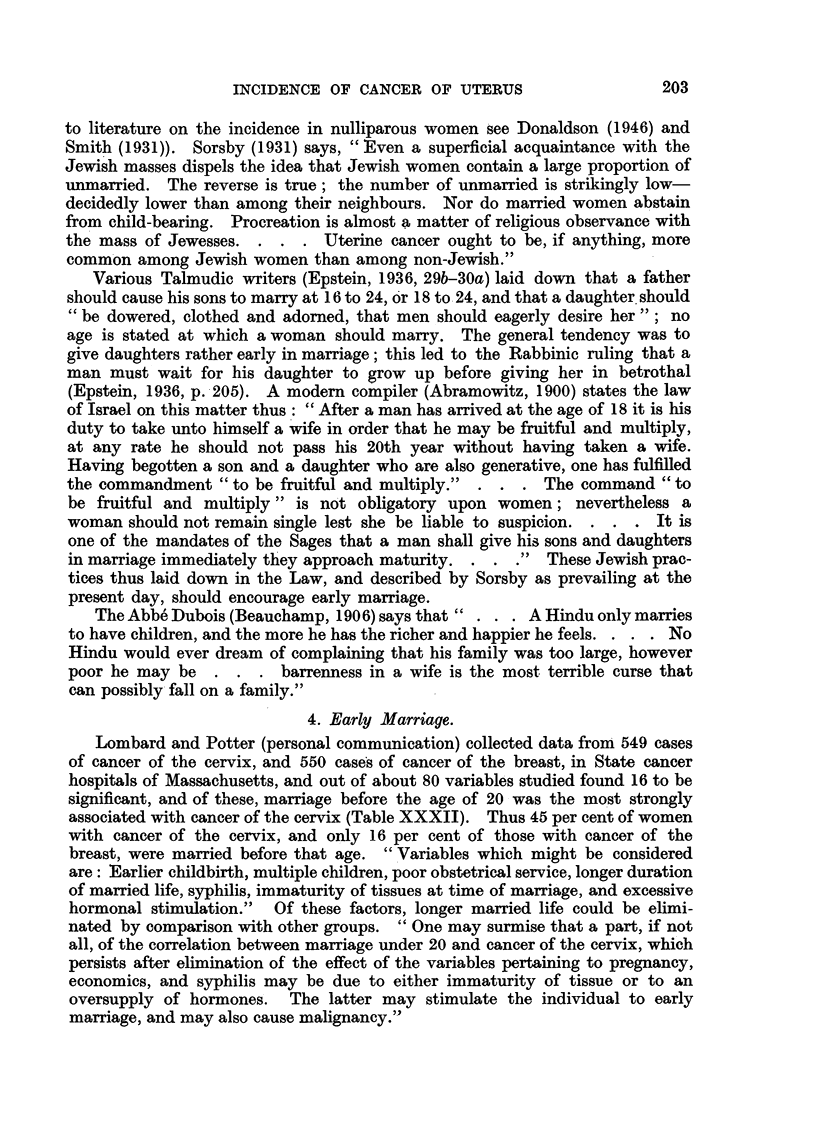

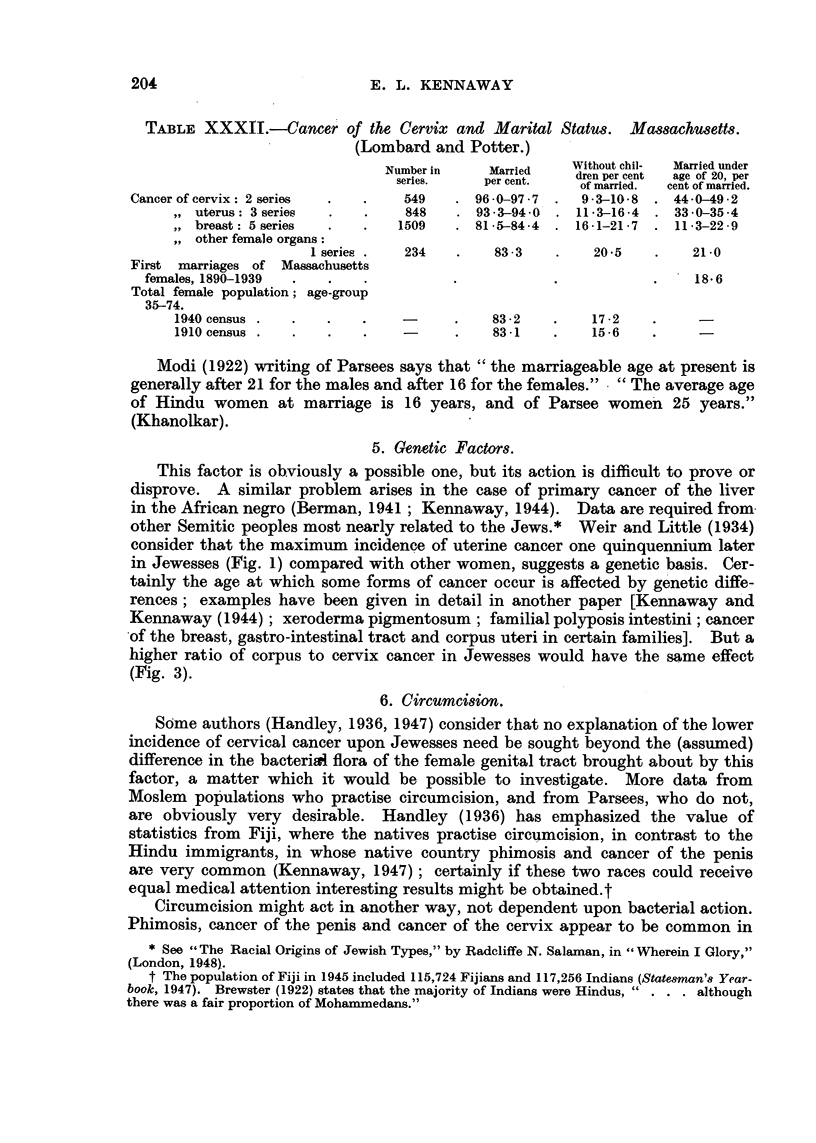

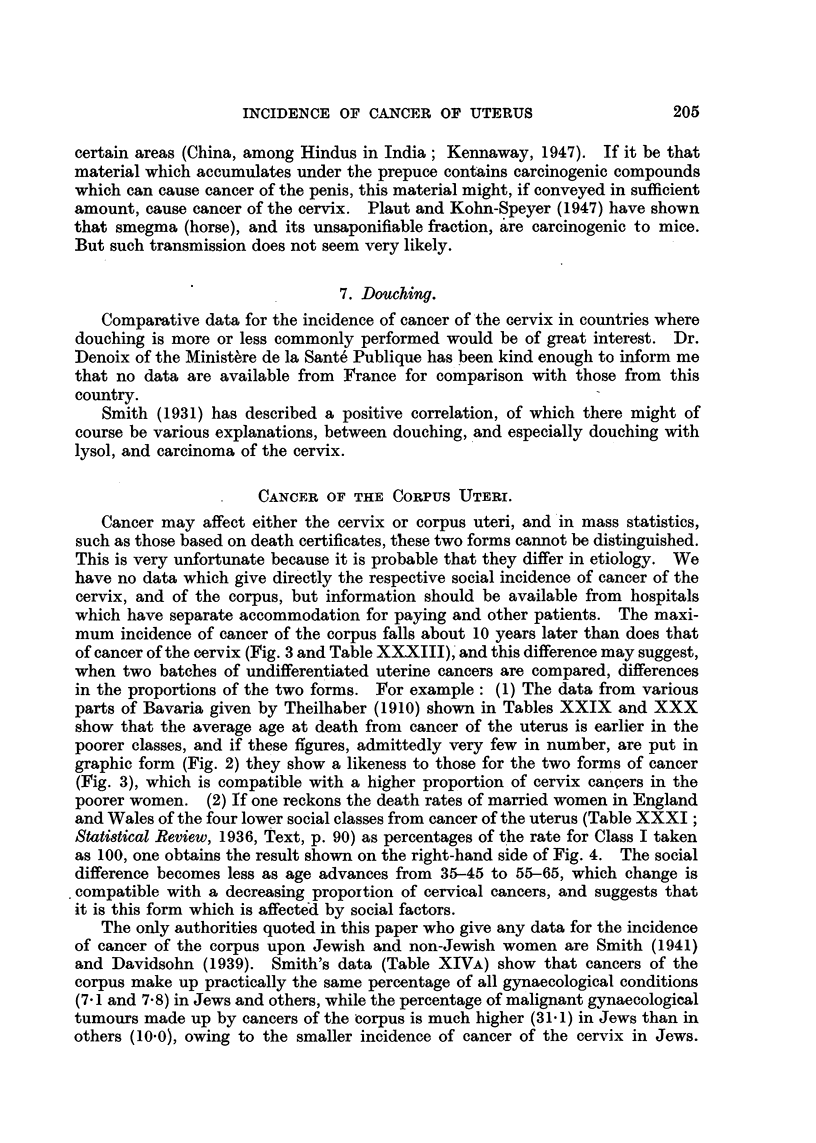

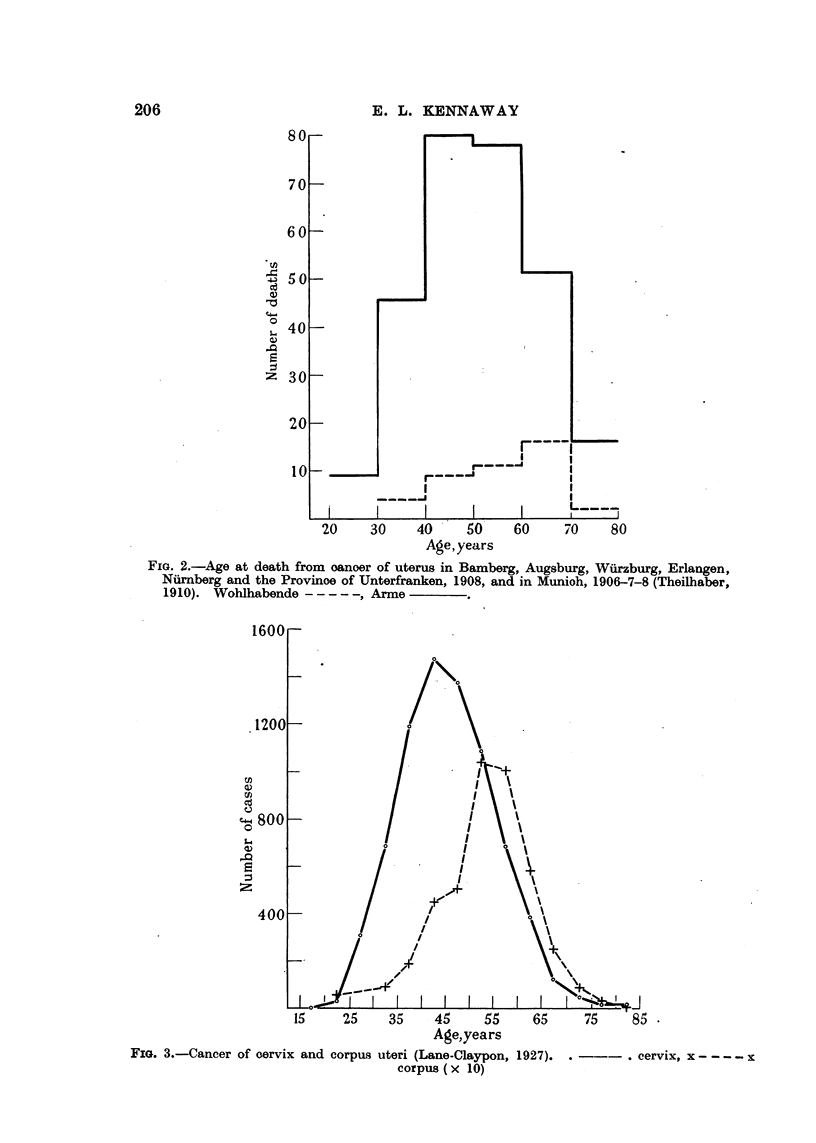

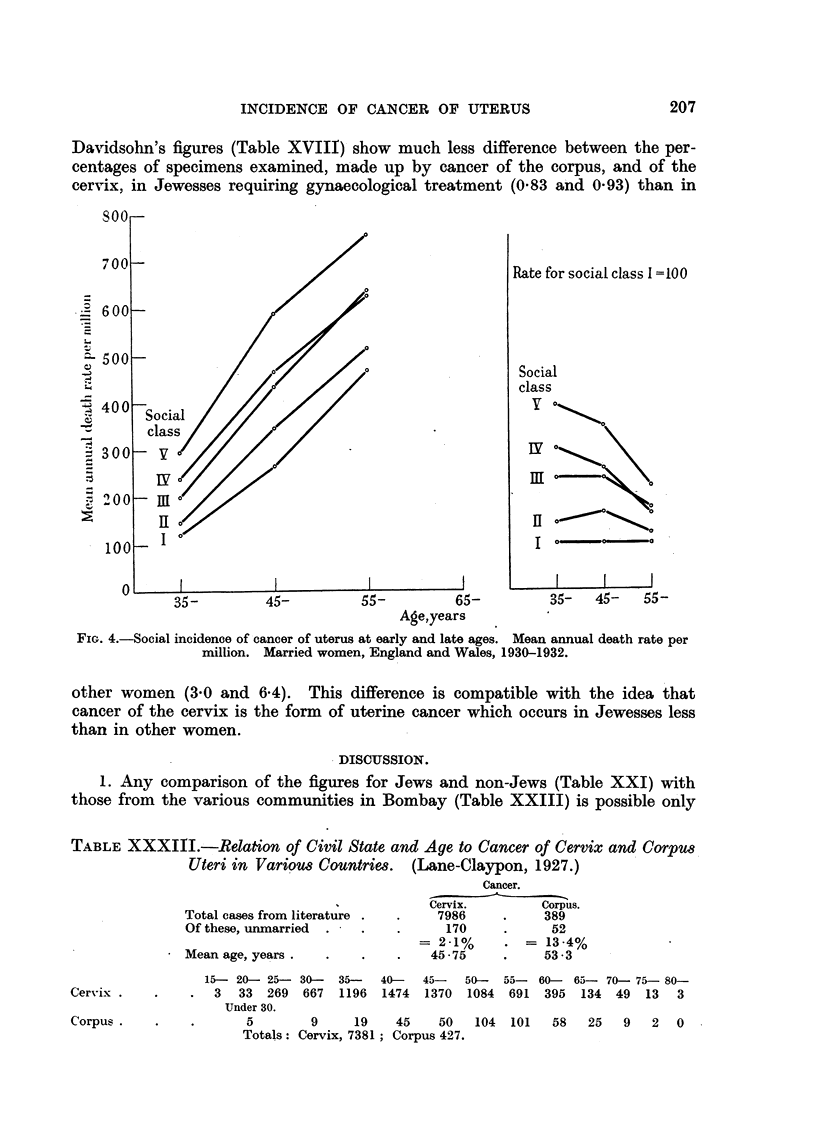

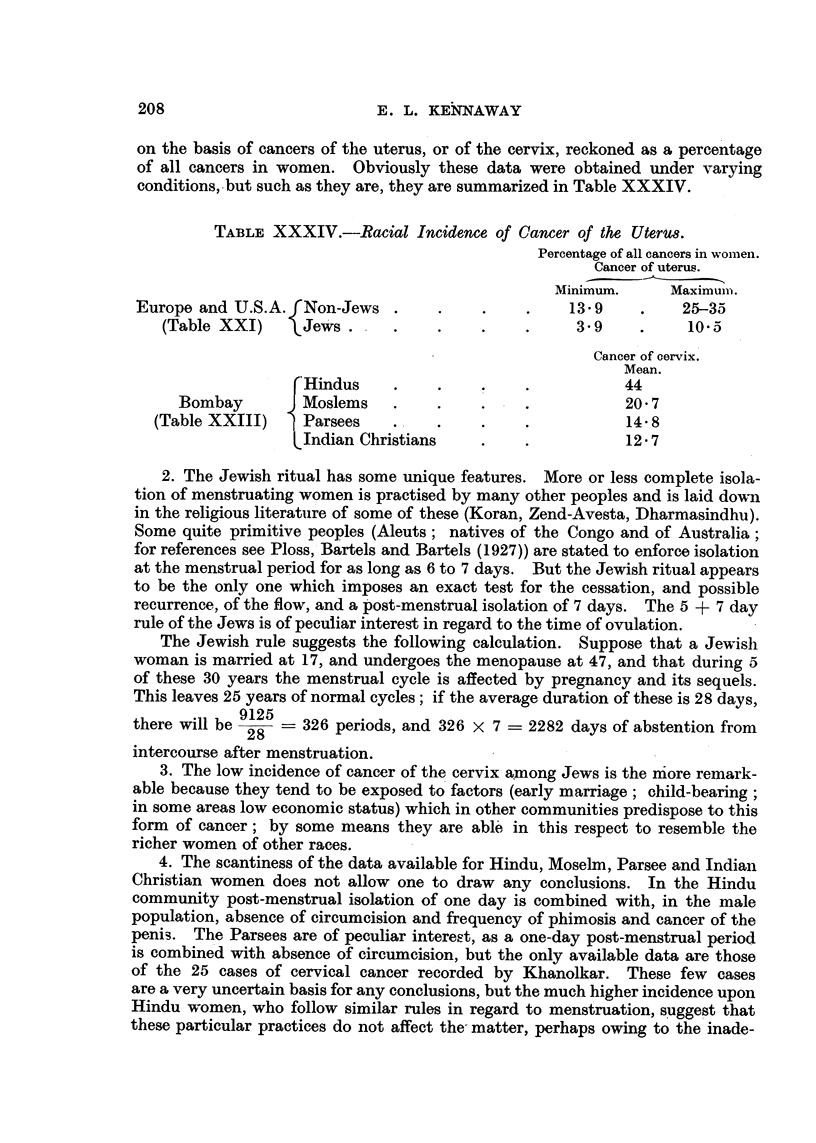

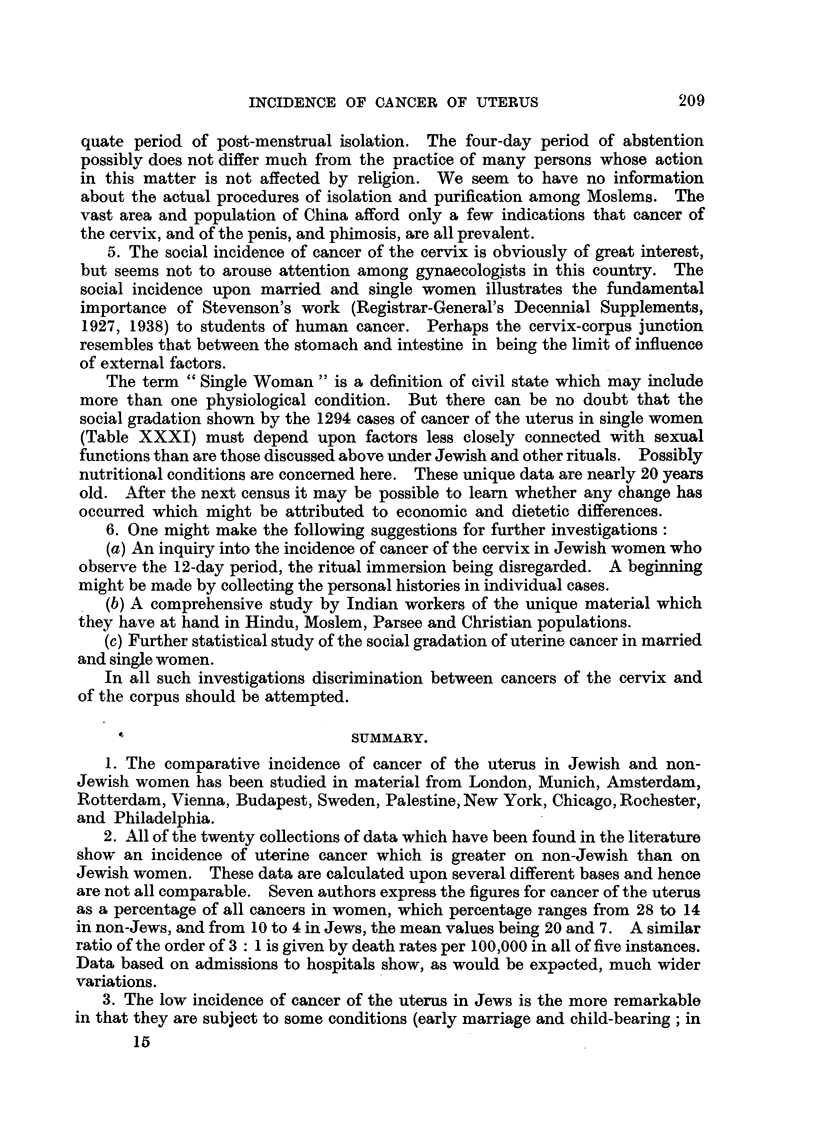

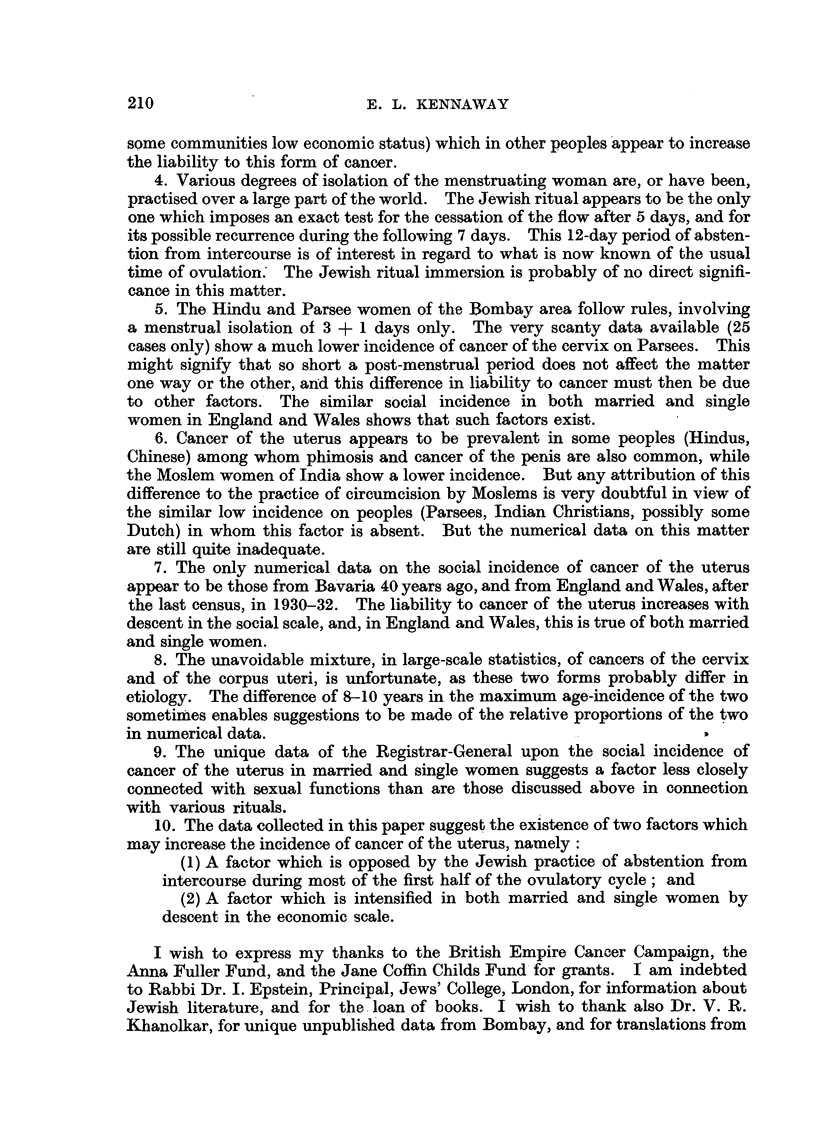

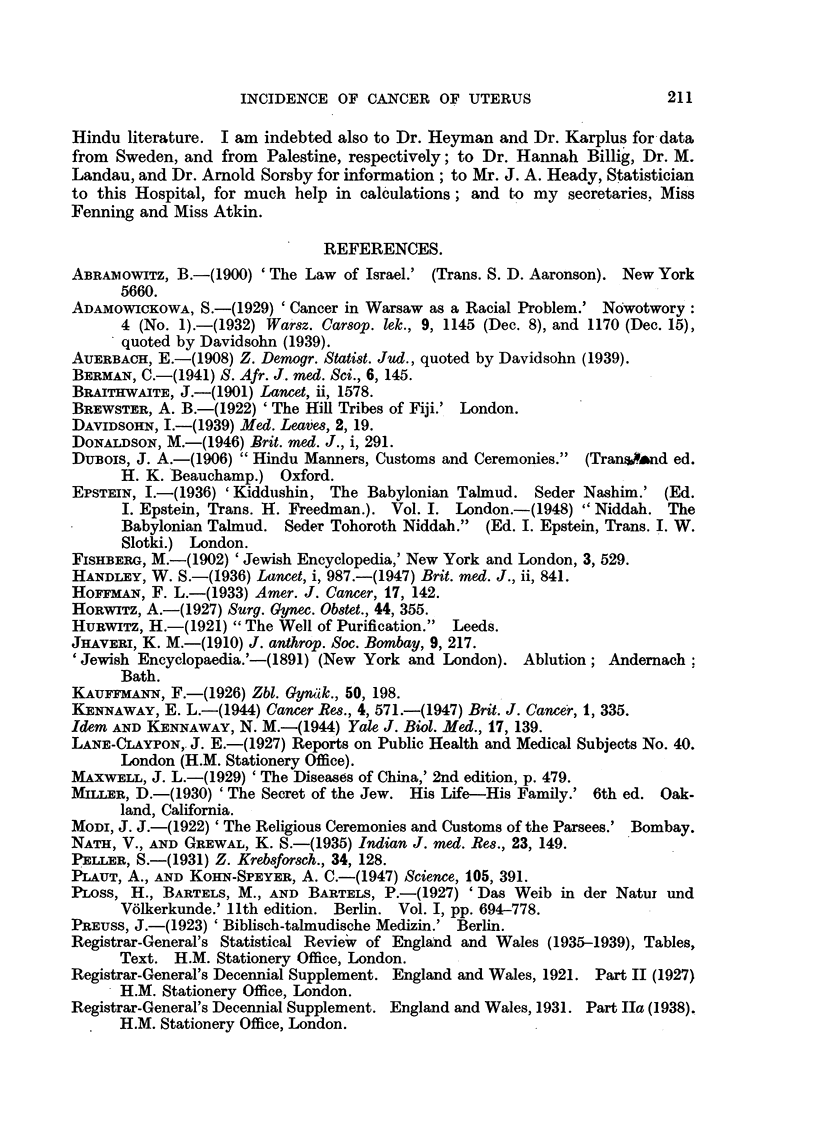

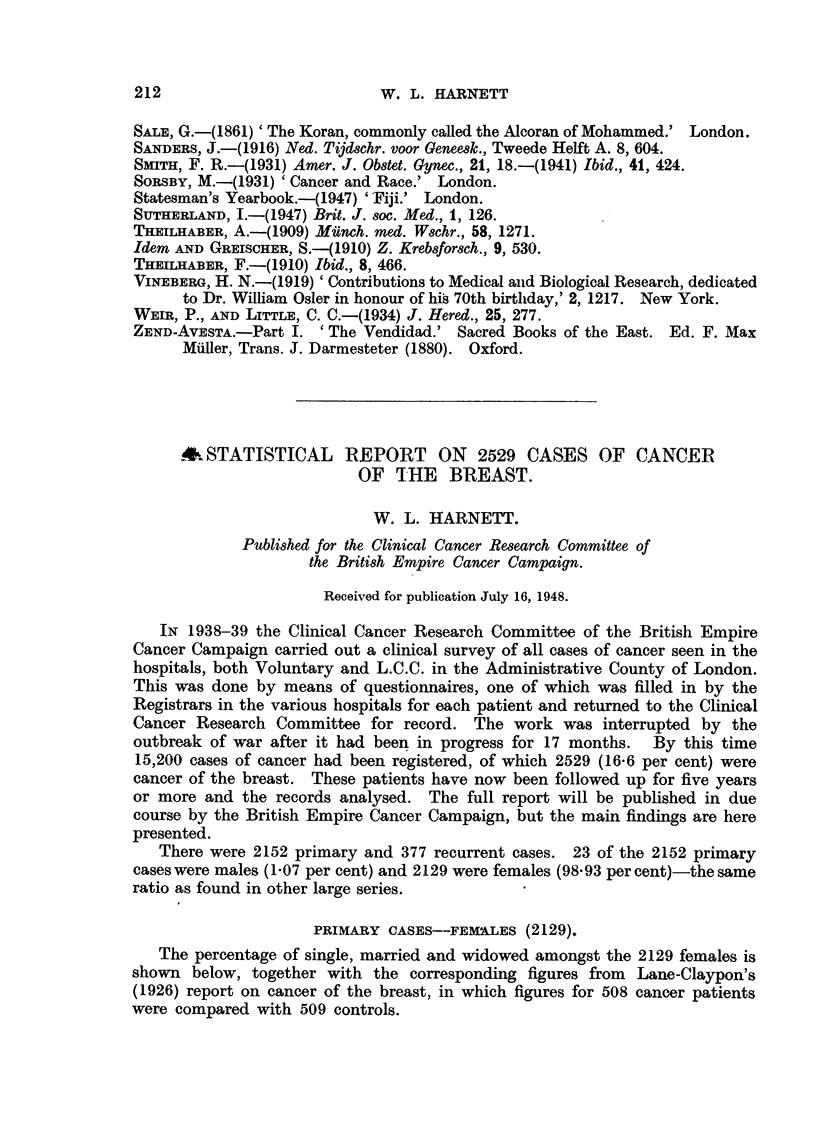

